# Panoramic analysis of the biological function and clinical value of SLC38A2 in human cancers: a study based on pan-cancer and single-cell analysis

**DOI:** 10.3389/fgene.2025.1658299

**Published:** 2025-09-17

**Authors:** Mao Liao, Yuqing Rao, Molan Li, Jiayang Guo, Kun Guo, Kaiyue Li, Rui Zheng, Yifan Liu, Qianyi Wang, Manni Wang, Duo Chen, Meng Zhang, Yongfeng Wang, Yanzong Zhao, Sheng Li

**Affiliations:** ^1^ The Second Hospital & Clinical Medical School, Lanzhou University, Lanzhou, China; ^2^ College of International Education, Henan University of Technology, Zhengzhou, China; ^3^ The First Hospital & Clinical Medical School, Lanzhou University, Lanzhou, China; ^4^ School of Stomatology, Lanzhou University, Lanzhou, China; ^5^ School of Life Science, Lanzhou University, Lanzhou, China; ^6^ The First People’s Hospital of Lanzhou City, Lanzhou, China

**Keywords:** cancer, slc38a2, glutamine, metabolic reprogramming, single-cell, prognosis

## Abstract

**Background:**

Glutamine metabolic reprogramming is a hallmark of tumor progression and is highly correlated with poor clinical outcomes. The excessive uptake of glutamine by tumor cells is a key factor contributing to widespread invasion, metastasis, and immune suppression. SLC38A2, an amino acid transporter widely expressed on the surface of tumor cells, has not been thoroughly studied regarding its function and prognostic significance in tumor progression. Our objective is to employ bioinformatics methods to conduct a comprehensive and in-depth analysis of SLC38A2 across various cancers, aiming to elucidate its role and prognostic value in tumor biology.

**Methods:**

By comprehensively incorporating gene expression and clinical data from the TCGA tumor database, GTEx database, Human Protein Atlas, and GEO database, we analyzed the expression profile, mutations, and established prognostic models for SLC38A2 across various cancers. Additionally, we investigated the enrichment of SLC38A2 at the single-cell level in 12 types of cancer and analyzed its temporal expression patterns in different cell subgroups in breast and pancreatic cancer. We also studied the correlation between SLC38A2 and glutathione metabolism.

**Results:**

Compared to normal tissues, SLC38A2 exhibits significant differential expression in 15 types of cancer and serves as a prognostic risk factor in BRCA (HR = 1.597, *p* < 0.05), LUAD (HR = 1.650, *p* < 0.01), MESO (HR = 2.007, *p* < 0.05), and PAAD (HR = 1.761, *p* < 0.05), while acting as a protective factor in KIRC (HR = 0.625, *p* < 0.05). Furthermore, SLC38A2 is positively correlated with tumor and stromal cells, negatively correlated with immune cell infiltration, and associated with immune exhaustion. In BRCA, SLC38A2 is highly expressed during early differentiation of malignant and stromal cells, and enriched in late differentiation of immune cells. Moreover, the expression of SLC38A2 shows a general positive correlation with glutathione metabolism genes in BRCA, LUAD, MESO, and PAAD, demonstrating diagnostic value.

**Conclusion:**

SLC38A2 shows widespread changes in expression patterns within tumor tissues, making it an effective diagnostic and prognostic biomarker. It is enriched in malignant cells and tumor-infiltrating stromal cells, while negatively correlated with the infiltration of many cells involved in anti-tumor immunity. Targeting SLC38A2 presents a viable therapeutic strategy by inhibiting glutamine competition and relieving immune suppression in the tumor microenvironment.

## Graphical Abstract

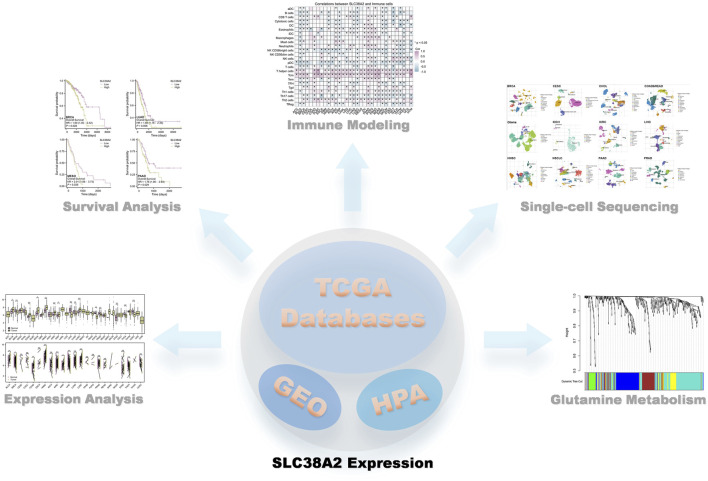

## 1 Introduction

Malignant tumors, also known as cancer, remain the leading non-communicable disease threatening human health. Although molecular targeted therapy and immunotherapy have brought paradigm shifts in cancer management, the heterogeneity factors within the tumor microenvironment (TME) challenge standardized treatment strategies and may render these methods ineffective (Kaur et al., 2023; Liu et al., 2023; Bejarano et al., 2021). Recently, the role of nutrient metabolism in TME in tumorigenesis and progression has received widespread attention (Zhu et al., 2022; Vitale et al., 2019; Li et al., 2021a). As a crucial component of tumor metabolic reprogramming, abnormal amino acid metabolism is a hallmark of many malignant tumors and is associated with tumor cell proliferation, invasion, and metastasis (Li and Zhang, 2016; Li and Zhang, 2024; Lieu et al., 2020). Due to the upregulation of metabolic activity in tumor cells, when the tricarboxylic acid cycle and anaerobic glycolysis (also known as the Warburg effect) cannot meet the energy demands of tumor cells, amino acid metabolism is activated as an alternative fuel source (Keenan and Chi, 2015). Additionally, some tumor cells excessively uptake amino acids in TME, creating nutritional competition with other cells in the stroma, which can lead to the deterioration of the microenvironment (Lieu et al., 2020). In the reprogramming of amino acid metabolism in tumor cells, glutamine plays a crucial role. Compared to normal cells, molecules involved in the regulation of glutamine metabolism in tumor cells exhibit widespread abnormal expression, such as Myc (Gao et al., 2009), p53 (Tran et al., 2017), Ras (Dilshara et al., 2017), and hypoxia-inducible factor (HIF) (Faubert et al., 2014). Glutamine not only provides energy and raw materials for macromolecule synthesis in tumor cells, but its metabolic pathways also generate glutathione (GSH) and nicotinamide adenine dinucleotide phosphate (NADPH). These metabolites help eliminate excess reactive oxygen species (ROS) within the tumor cells, thereby supporting their survival (Niu et al., 2021; Kennedy et al., 2020).

**FIGURE 1:**
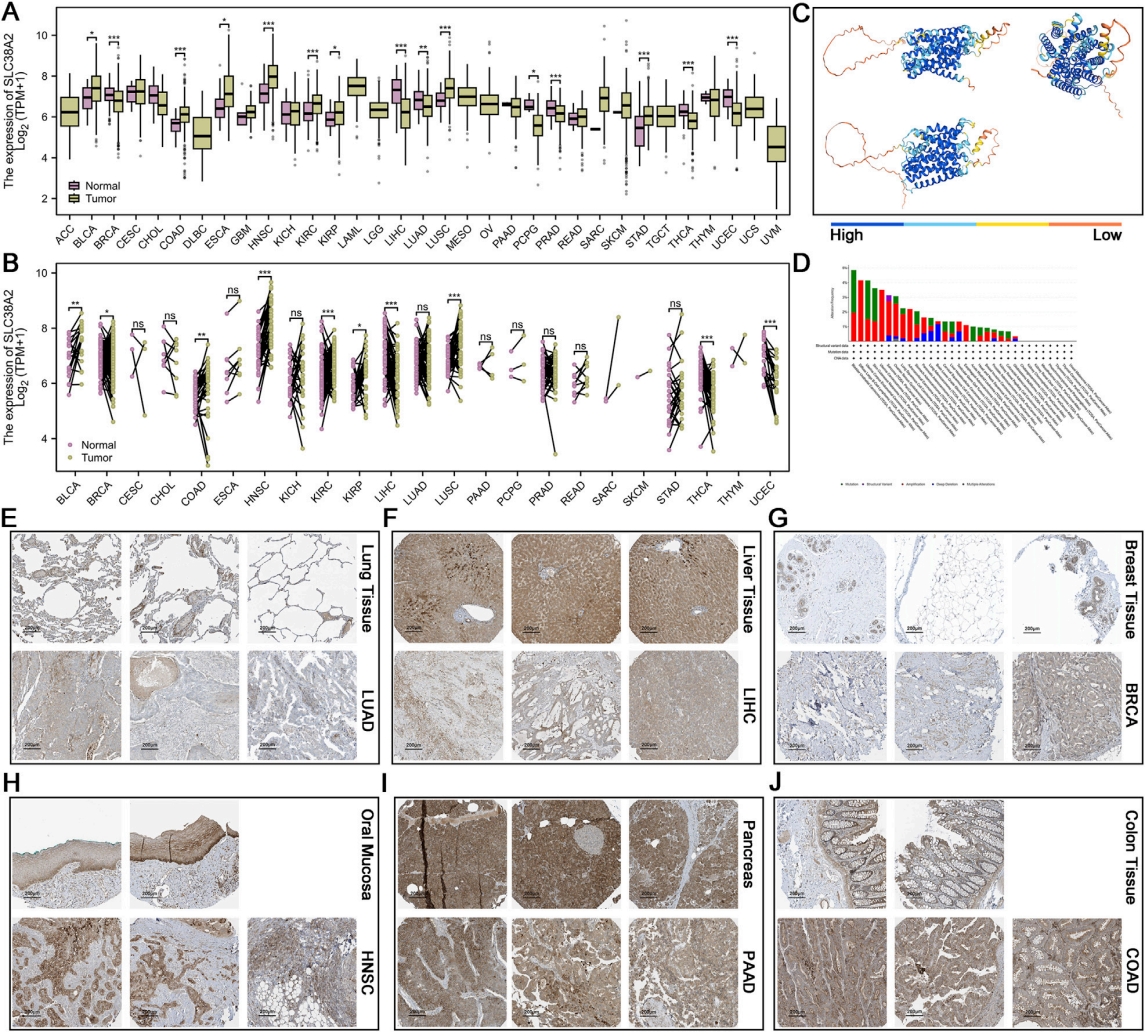
**(A)** Expression differences of the SLC38A2 gene in cancer tissues and normal tissues (unpaired samples). **(B)** Expression differences of the SLC38A2 gene in cancer tissues and normal tissues (paired samples). **(C)** Predicted protein structure of SLC38A2, with blue indicating high confidence. **(D)** Gene mutations of SLC38A2 in various types of cancer. **(E)** Expression of SLC38A2 in normal lung alveoli and lung adenocarcinoma tissues. **(F)** Expression of SLC38A2 in normal liver and hepatocellular carcinoma tissues. **(G)** Expression of SLC38A2 in normal breast and breast cancer tissues. **(H)** Expression of SLC38A2 in normal oral epithelial tissue and head and neck cancer (oral squamous cell carcinoma) tissue. **(I)** Expression of SLC38A2 in normal pancreatic tissue and pancreatic cancer tissue. **(J)** Expression of SLC38A2 in normal colon tissue and colon cancer tissue.

Recent studies indicate that tumor cells competitively sequester glutamine from the TME, affecting the activity of other cells in the tumor stroma and thereby influencing anti-tumor immune responses (Koppula et al., 2021; Wang et al., 2024; Leone et al., 2019). For instance, in competitive glutamine uptake in kidney renal clear cell carcinoma (KIRC), the deprivation of extracellular glutamine activates HIF-1α, leading to the induction of tumor-infiltrating macrophages to secrete IL-23. This further promotes the proliferation and activation of regulatory T cells (Tregs), ultimately weakening the anti-tumor immune response (Fu et al., 2019). Summarizing the aforementioned points, the enrichment of glutamine transporters may serve as a marker of tumor progression and holds potential as a target for cancer therapy. Solute carrier family 38 member A2 (SLC38A2) is a glutamine transporter closely linked to the high accumulation of glutamine within tumor cells (Figure 1C) (Menchini and Chaudhry, 2019; Broer et al., 2016). Our research hypothesizes that SLC38A2 functions as a broadly significant immune and prognostic biomarker in cancer, potentially serving as a target for therapeutic intervention. This hypothesis is supported by evidence from several studies. For example, Chuansheng et al. has demonstrated that the expression of SLC38A2 and the subsequent glutamine-mediated metabolic crosstalk between tumor cells and conventional dendritic cells type 1 (cDC1) form the basis of tumor immune evasion (Guo et al., 2023). Addintionally, several studies underscore the pivotal role of SLC38A2 in the clinical outcomes of various cancers. Mengsen et al. conducted a comprehensive 5-year pan-cancer cohort analysis, revealing that the overexpression of SLC38A2 is emblematic of cancer’s metabolic characteristics and correlates with diminished survival rates (Huang et al., 2020). Similarly, Liang et al. identified SLC38A2 as a prognostic risk factor in gastric cancer, associating it with immune infiltration and the activation of M2 macrophages (Zhu et al., 2023). Although these findings suggest an association between SLC38A2 and cancer prognosis, TME, and immunity, there are limitations. The primary gaps lie in the analysis of specific cancer types and biological effects, necessitating further research for validation.

Therefore, we integrated tumor gene and protein expression data from the TCGA, GTEx, Human Protein Atlas (HPA), and GEO databases. And by the using of bioinformatics methods, we comprehensively analyzed these data to elucidate the biological functions of SLC38A2 across various cancer types and its associations with tumor immunity and clinical prognosis.

## 2 Materials and methods

### 2.1 Cancer names and abbreviations

The cancer names and abbreviations involved in this study include Adrenocortical Carcinoma (ACC), Bladder Urothelial Carcinoma (BLCA), Breast Invasive Carcinoma (BRCA), Cervical Squamous Cell Carcinoma and Endocervical Adenocarcinoma (CESC), Cholangiocarcinoma (CHOL), Colon Adenocarcinoma (COAD), Lymphoid Neoplasm Diffuse Large B-cell Lymphoma (DLBC), Esophageal Carcinoma (ESCA), Glioblastoma Multiforme (GBM), Head and Neck Squamous Cell Carcinoma (HNSC), Kidney Chromophobe (KICH), Kidney Renal Clear Cell Carcinoma (KIRC), Kidney Renal Papillary Cell Carcinoma (KIRP), Acute Myeloid Leukemia (LAML), Brain Lower Grade Glioma (LGG), Liver Hepatocellular Carcinoma (LIHC), Lung Adenocarcinoma (LUAD), Lung Squamous Cell Carcinoma (LUSC), Mesothelioma (MESO), Non-Small Cell Lung Cancer (NSCLC), Ovarian Serous Cystadenocarcinoma (OV), Pancreatic Adenocarcinoma (PAAD), Pheochromocytoma and Paraganglioma (PCPG), Prostate Adenocarcinoma (PRAD), Rectum Adenocarcinoma (READ), Sarcoma (SARC), Skin Cutaneous Melanoma (SKCM), Stomach Adenocarcinoma (STAD), Testicular Germ Cell Tumors (TGCT), Thyroid Carcinoma (THCA), Thymoma (THYM), Uterine Corpus Endometrial Carcinoma (UCEC), Uterine Carcinosarcoma (UCS), and Uveal Melanoma (UVM).

### 2.2 Data and sample information

The foundational data for this study were sourced from the TCGA and GTEx databases, comprising 15,901 normal or adjacent tissue samples and 10,201 tumor tissue samples. These include gene expression data for SLC38A2 and clinical data from patients. Additionally, single-cell sequencing data from the National Institutes of Health (NIH) were utilized, covering datasets GSE176078, GSE168652, GSE138709, GSE166555, GSE162631, GSE171306, GSE166635, GSE162025, GSE162708, and GSE141445. Moreover, data from the omicsdi database (EMTAB6149) and the NGDC database (CRA001160) were included. The study also employed immunohistochemistry slides and protein 3D structure data from the Human Protein Atlas (HPA).

For TCGA data, we downloaded and organized RNAseq data from 33 cancer projects processed by the STAR pipeline and extracted the data in TPM format. We discarded data without corresponding clinical information and converted it to log_2_(value + 1). For expression matrices downloaded from the GEO database, we used the ComBat_Seq function from the “sva” package to remove batch effects, preserving grouping information using the mod option.

### 2.3 Expression profile of SLC38A2 in 33 common types of cancer

From the TCGA expression database, we used the R packages “ggplot2”, “stats”, and “car” for data visualization, and apply the Wilcoxon rank sum test for statistical analysis.

### 2.4 Analysis of single-cell expression profile of SLC38A2

The scRNA-seq data for single-cell analysis was derived from 12 datasets, and the expression matrix transformation and batch effect correction were completed as described earlier. Subsequently, the “Seurat” package was used for quality control. Specifically, genes expressed in at least three cells and cells with at least 200 genes expressed were extracted for data initialization. During the filtering stage, cells with gene counts between 200 and 7,500 were retained, and cells with mitochondrial gene proportions exceeding 20% were removed (Zhang et al., 2024). The “FindVariableFeatures” function was used to select 3,000 highly variable genes for analysis, the “FindNeighbors” function was used to construct a KNN graph based on the top 30 PCs, and UMAP was employed for visualization. For cell annotation, the “FindAllMarkers” function was used to identify differentially expressed genes for each cluster and perform automatic annotation. Additionally, the “FeaturePlot” function was used to label and visualize the expression of SLC38A2.

### 2.5 Analysis of correlation between SLC38A2 and clinical outcomes

For the forest plot of SLC38A2 across various cancers, select OS (Overall Survival), DSS (Disease-Specific Survival), and PFI (Progression-Free Interval) as outcome variables. Use the “survival” package to test the proportional hazards assumption and perform univariate Cox regression analysis.

For multivariate Cox regression analysis, we use Overall Survival (OS) as the outcome variable. The default clinical indicators included are pathologic M stage, pathologic T stage, pathologic N stage, gender, and age. For cancer types without TNM staging, we use the WHO classification standards. The proportional hazards assumption is tested using the survival package, followed by multivariate Cox regression analysis. Variables meeting the set p-value threshold in the univariate analysis are included in the multivariate Cox model construction. Visualize the forest plot using the “ggplot2” package. For survival curves, use the “survminer” and “ggplot2” packages for visualization.

### 2.6 Correlation between SLC38A2 and TME

To analyze the correlation between SLC38A2 expression and immune infiltration matrix data across 33 types of cancer in the TCGA database, the ssGSEA algorithm provided by the R package “GSVA” was utilized. This involved calculating immune infiltration using markers of 24 immune cell types. Additionally, the “estimate” package was used to compute stromal and immune scores. Visualization was performed using the “ggplot2” package.

### 2.7 Pseudotemporal analysis of SLC38A2 expression

Based on previous cell annotation results, divide the single-cell data in BRCA and PAAD into three major cell groups: malignant cells, immune cells, and stromal cells for independent trajectory analysis. Use the R package “Monocle3” for pseudotemporal analysis, apply “learn graph” to learn cell development trajectories, and use “order cells” to set the root node based on biological markers. Use “graph test” to identify differentially expressed genes along the trajectory.

### 2.8 Weighted gene co-expression network analysis (WGCNA)

We obtained 580 samples from the LUAD cohort (58 normal tissues and 522 tumor tissues) and 503 samples from the COAD cohort (41 normal tissues and 462 tumor tissues) for differential analysis based on the expression profiles of 947 metabolism-related genes. Significant differentially expressed genes were identified using the DESeq2 algorithm. In COAD, 518 out of 947 metabolism-related genes (54.7%) were differentially expressed between normal (41 samples) and colorectal cancer (462 samples) tissues, with 253 genes upregulated and 265 genes downregulated in tumors. In LUAD, 244 out of 947 metabolism-related genes (25.7%) were differentially expressed between normal (58 samples) and lung adenocarcinoma (522 samples) tissues, with 160 genes upregulated and 84 genes downregulated in tumors.

Subsequently, WGCNA was performed on the 244 and 518 differentially expressed genes in the two cancers, respectively. A scale-free topology network was constructed based on the soft threshold power, and co-expression modules were identified using the dynamic tree cut algorithm. After calculating module eigengenes, their Pearson correlation with clinicopathological features (T stage, N stage, pathological stage, and overall survival) was assessed. The clusterProfiler was used for KEGG pathway enrichment analysis of key modules, analyzing the blue module in LUAD and the yellow module in COAD.

Based on the tumor samples from both, we first performed molecular subtyping using consensus clustering (ConsensusClusterPlus R package), determining the optimal number of clusters k = 3 through cumulative distribution function (CDF) analysis. Differential expression analysis (DESeq2 R package) was then conducted for the three subtypes (C1/C2/C3), and Kaplan-Meier survival analysis (survival R package) was used to evaluate prognostic differences among subtypes.

### 2.9 Drug sensitivity analysis

We collected the half-maximal inhibitory concentration (IC_50_) values of 265 small molecules and their corresponding mRNA gene expression data from 860 cell lines in the Genomics of Drug Sensitivity in Cancer (GDSC). Similarly, we gathered IC_50_ values of 481 small molecules and their corresponding mRNA gene expression data from 1,001 cell lines in the Cancer Therapeutics Response Portal (CTRP). We then integrated the mRNA expression and drug sensitivity data. Subsequently, we performed a Pearson correlation analysis to determine the correlation between SLC38A2 expression and IC_50_ values. Finally, a volcano plot was created using the “ggplot2” package, with the horizontal axis representing the correlation coefficients and the vertical axis representing the adjusted p-values.

### 2.10 Statistical analysis

The Wilcoxon test was used to calculate expression differences between groups. Log-rank and COX regression were employed to examine the significance of survival curve differences between high and low SLC38A2 expression groups. Spearman correlation analysis was used to assess the correlation between SLC38A2 expression and the TME (including immune score, stromal score, and immune cell infiltration). All data were log-transformed. All p-values were adjusted for false discovery using the Benjamini–Hochberg procedure. Significant associations in analyses required an FDR-adjusted p-value (q-value) < 0.05, and a p-value of less than 0.05 was considered statistically significant.

## 3 Results

### 3.1 Panoramic expression profile of SLC38A2 in cancer

The results of the unpaired sample differential analysis indicate that SLC38A2 exhibits differential expressions in 14 types of cancer tissues compared to adjacent non-cancerous tissues. Specifically, SLC38A2 is overexpressed in eight types of cancer which include BLCA (*p* < 0.05), COAD (*p* < 0.001), ESCA (*p* < 0.05), HNSC (*p* < 0.001), KIRC (*p* < 0.001), KIRP (*p* < 0.05), LUSC (*p* < 0.001), and STAD (*p* < 0.001). Conversely, it is underexpressed in six types of cancer that include BRCA (*p* < 0.001), LIHC (*p* < 0.001), LUAD (*p* < 0.01), PRAD (*p* < 0.001), THCA (*p* < 0.001) and UCEC (*p* < 0.001) (Figure 1A). In paired sample analysis, SLC38A2 shows differential expressions in 10 types of cancer, including BLCA (*p* < 0.01), COAD (*p* < 0.01), HNSC (*p* < 0.001), KIRC (*p* < 0.001), KIRP (*p* < 0.05), LUSC (*p* < 0.001), BRCA (*p* < 0.05), LIHC (*p* < 0.001), THCA (*p* < 0.001), and UCEC (*p* < 0.001). The first six cancers correspond to overexpression as observed in the unpaired sample analysis, while the last four correspond to underexpression (Figure 1B).

Immunohistochemistry results indicate that SLC38A2 expression is significantly lower in LIHC and PAAD compared to normal liver and pancreatic tissues. In contrast, its expression is higher in COAD compared to normal colonic epithelium. Additionally, the expression of SLC38A2 in lung, breast, and oral epithelial tissues, as well as their corresponding cancers (LUAD, BRCA and HNSC) were also examined (Figures 1E–J).

The mutation analysis results indicate that the main types of mutations for SLC38A2 in cancer are amplification, mutation, and deep deletion. The top three cancers with the highest probability of these mutations are BLCA, DLBC, and UCEC (Figure 1D).

### 3.2 SLC38A2 is an independent risk factor of cancer

**FIGURE 2:**
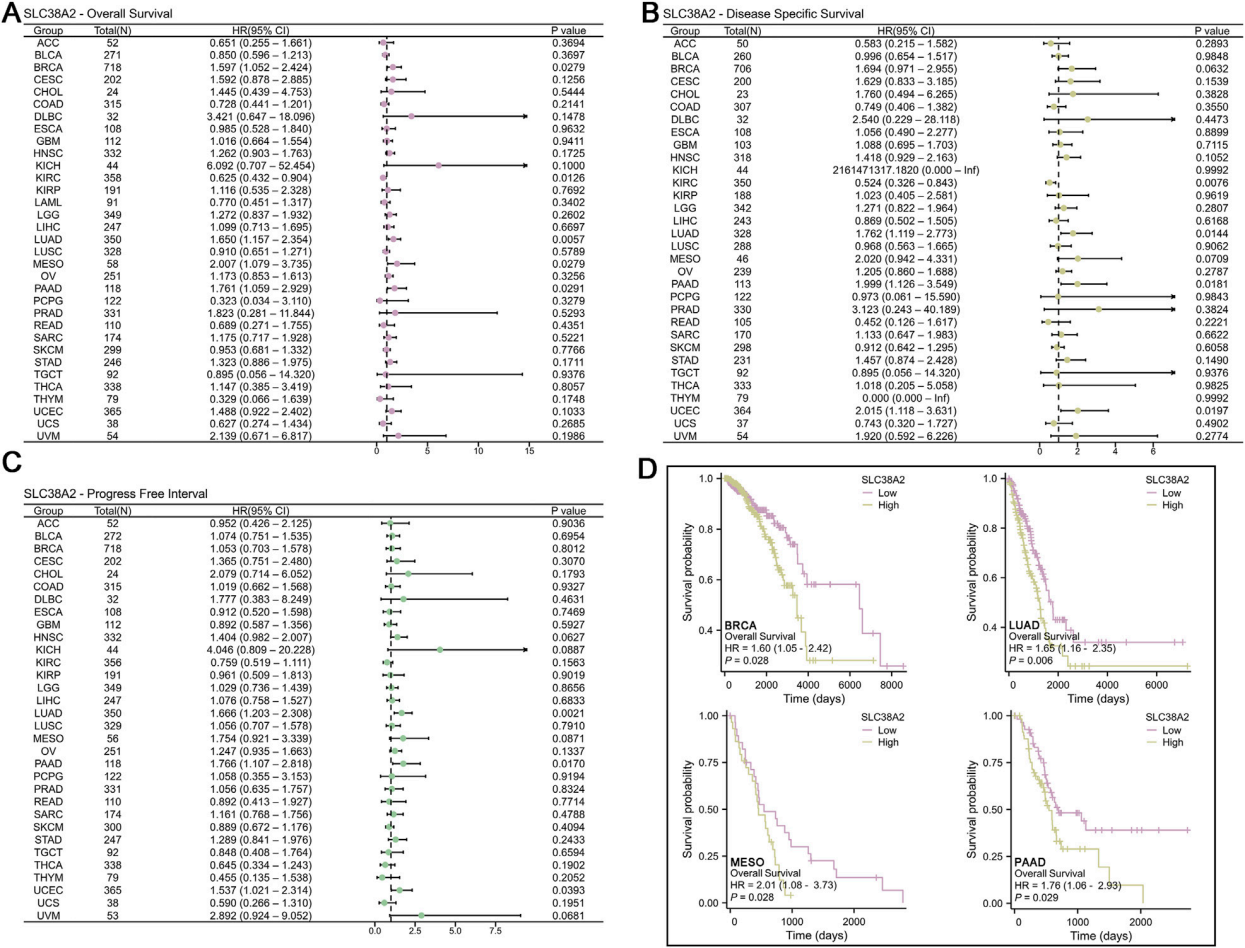
**(A)** Forest plot of SLC38A2 across various cancers using Overall Survival as the evaluation metric. **(B)** Forest plot of SLC38A2 across various cancers using Disease-Specific Survival as the evaluation metric. **(C)** Forest plot of SLC38A2 across various cancers using Progression-Free Interval as the evaluation metric. **(D)** Survival curves for patients with BRCA, LUAD, MESO, and PAAD, categorized by high and low expression of SLC38A2.

Using overall survival (OS) as the evaluation metric, SLC38A2 serves as a risk factor for BRCA (HR = 1.597, *p* < 0.05), LUAD (HR = 1.650, *p* < 0.01), MESO (HR = 2.007, *p* < 0.05), and PAAD (HR = 1.761, *p* < 0.05), while acting as a protective factor for KIRC (HR = 0.625, *p* < 0.05) (Figure 2A). For disease-specific survival (DSS), SLC38A2 is a risk factor for LUAD (HR = 1.762, *p* < 0.05) and PAAD (HR = 1.999, *p* < 0.05), and a protective factor for KIRC (HR = 0.524, *p* < 0.01) (Figure 2B). In terms of progression-free interval (PFI), SLC38A2 is a risk factor for LUAD (HR = 1.666, *p* < 0.01) and PAAD (HR = 1.766, *p* < 0.05) (Figure 2C). Survival curve results indicate that high expression of SLC38A2 is associated with shorter overall survival in BRCA, LUAD, MESO, and PAAD, suggesting a correlation with poor prognosis (Figure 2D). Additionally, the results of the multivariate Cox regression further confirmed that SLC38A2 acts as an independent risk factor in these cancers (Supplementary Material 1).

### 3.3 The correlation between SLC38A2 and immune microenvironment

**FIGURE 3:**
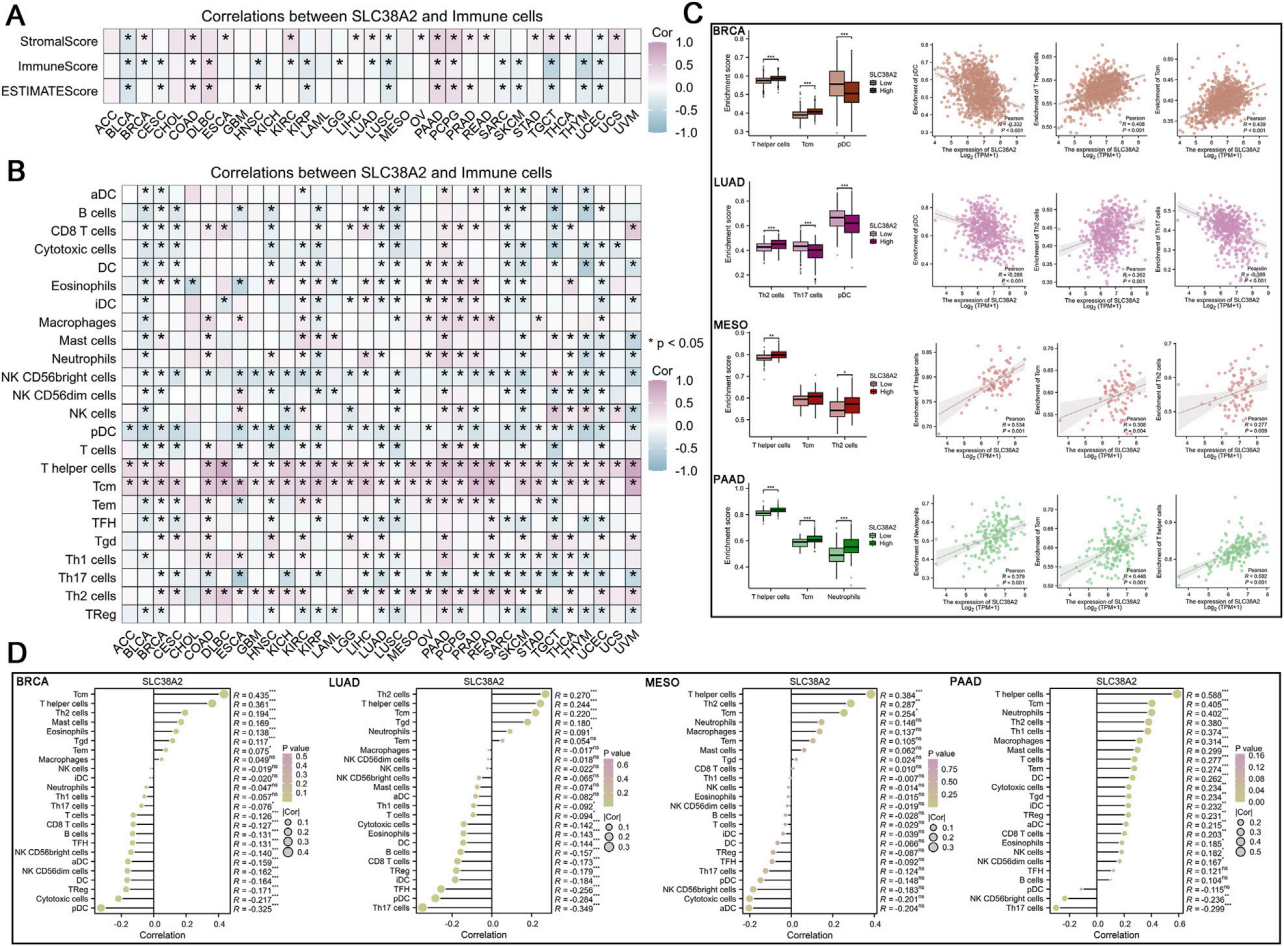
**(A)** Correlation between SLC38A2 expression and immune scores and stromal scores across various cancers using the Estimate algorithm. **(B)** Correlation between SLC38A2 expression and immune cell infiltration across various cancers using the ssGSEA algorithm. **(C)** Correlation between SLC38A2 expression and immune cell infiltration in BRCA, LUAD, MESO, and PAAD, grouped by high and low expression of SLC38A2. **(D)** Correlation between SLC38A2 expression and immune cell infiltration in BRCA, LUAD, MESO, and PAAD, with statistical significance considered at *p* < 0.05.

The analysis results from the Estimate algorithm indicate that the expression of SLC38A2 is positively correlated with stromal scores in most cancer types, including BRCA, COAD, ESCA, KIRC, LIHC, LUAD, OV, PAAD, PCPG, PRAD, READ, STAD, TGCT, THCA, and UCS. In contrast, for immune scores, SLC38A2 exhibits a negative correlation pattern in cancers such as BLCA, BRCA, CESC, HNSC, KIRC, KIRP, LGG, LUAD, LUSC, SARC, SKCM, TGCT, THYM, and UCEC (Figure 3A).

The analysis results from the ssGSEA algorithm indicate that the expression of SLC38A2 is generally negatively correlated with immune cells in most cancers. This includes aDCs, B cells, CD8^+^ T cells, cytotoxic cells, DCs, NK CD56bright cells, NK CD56dim cells, NK cells, pDC, T cells, TFH, Th17 cells, and Tregs. Additionally, SLC38A2 shows a generally positive correlation with certain immune cells, such as T helper cells, Tcm, Tem, Tgd and Th2 cells (Figure 3B). Further analysis was conducted on the correlation between SLC38A2 and infiltrating immune cells in four types of cancer which include BRCA, LUAD, MESO, and PAAD. The results indicate that SLC38A2 shows a moderate positive correlation with infiltrating Tcm (R = 0.435, *p* < 0.001) and T helper cells (R = 0.361, *p* < 0.001) in BRCA, while it exhibits a moderate negative correlation with pDCs (R = −0.325, *p* < 0.001) in BRCA. Additionally, SLC38A2 is moderately negatively correlated with infiltrating Th17 cells (R = −0.349, *p* < 0.001) in LUAD. In MESO, SLC38A2 demonstrates a moderate positive correlation with infiltrating T helper cells (R = 0.384, *p* < 0.001). In PAAD, SLC38A2 shows a strong positive correlation with infiltrating T helper cells (R = 0.588, *p* < 0.001) and a moderate positive correlation with infiltrating Tcm (R = 0.405, *p* < 0.001), Neutrophils (R = 0.402, *p* < 0.001), Th2 cells (R = 0.380, *p* < 0.001), Th1 cells (R = 0.374, *p* < 0.001), and Macrophages (R = 0.314, *p* < 0.001) (Figure 3D).

Additionally, we investigated the relationship between high and low expression of SLC38A2 and immune cell infiltration. In BRCA, the subgroup with high SLC38A2 expression corresponds to higher infiltration of T helper cells (*p* < 0.001) and Tcm (*p* < 0.001), and lower infiltration of pDCs (*p* < 0.001). In LUAD, the high SLC38A2 expression subgroup corresponds to higher infiltration of Th2 cells (*p* < 0.001) and lower infiltration of Th17 cells (*p* < 0.001) and pDCs (*p* < 0.001). In MESO, the high SLC38A2 expression subgroup is associated with increased infiltration of T helper cells (*p* < 0.01), and Th2 cells (*p* < 0.05). Finally, in PAAD, the subgroup with high SLC38A2 expression corresponds to greater infiltration of T helper cells (*p* < 0.001), Tcm (*p* < 0.001), and Neutrophils (*p* < 0.001) (Figure 3C).

### 3.4 Expression of SLC38A2 in single-cell subpopulation

**FIGURE 4:**
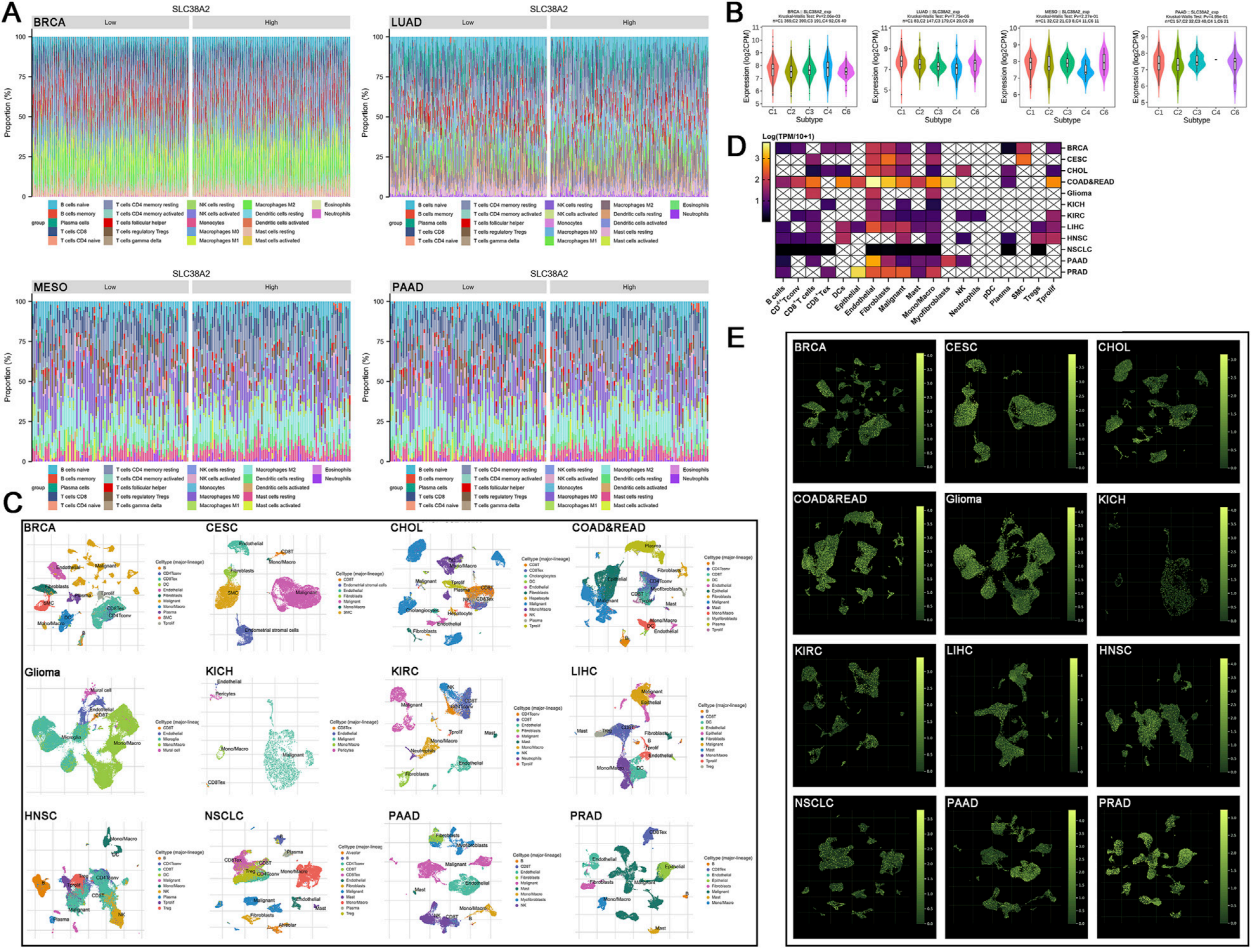
**(A)** Immune cell subtypes in BRCA, LUAD, MESO, and PAAD, grouped by high and low expression of SLC38A2. **(B)** Expression of SLC38A2 in different tumor immune subtypes. **(C)** t-SNE clustering of single-cell sequencing datasets from 12 types of cancer. **(D)** Expression of SLC38A2 in different cell subtypes, with expression levels converted to Log(TPM/10+1) format. **(E)** Expression of SLC38A2 in different cell subtypes, with higher green fluorescence intensity indicating higher expression levels.

By performing dimensionality reduction and clustering on high-throughput sequencing datasets from 12 types of cancer, we categorized the cells within the samples into the following subpopulations: Alveolar, B cells, Cholangiocytes, CD4^+^ Tconv, CD8^+^ T cells, CD8^+^ Tex, Dendritic Cells (DCs), Endothelial, Epithelial, Endometrial stromal cells, Fibroblasts, Hepatocytes, Monocytes/Macrophages (Mono/macro), Mast cells, Myofibroblasts, Microglia, Mural cells, Natural Killer (NK) cells, Neutrophils, Plasma cells, Pericytes, Smooth Muscle Cells (SMC), Trpolif and so on (Figures 4A,C).

We found that SLC38A2 is expressed across various cell lineages. Notably, there is a certain level of enrichment of SLC38A2 in tumor-associated stromal cells, such as epithelial cells, endothelial cells, and fibroblasts. In contrast, the expression of SLC38A2 is relatively low in immune-related cells (Figures 4D,E). Additionally, we discovered that SLC38A2 expression is associated with tumor immune subtypes. In BRCA, SLC38A2 is expressed at higher levels in tumors with C1 and C4 immune subtypes, while lower levels are observed in C2, C3, and C6 subtypes. In LUAD, SLC38A2 shows higher expression in C1 and C6 immune subtypes, with lower expression in C2, C3, and C4 subtypes. In MESO, higher expression is seen in C1, C3, and C6 immune subtypes, and lower expression in C2 and C4 subtypes. In PAAD, SLC38A2 is more highly expressed in C1, C3, and C6 subtypes, with reduced expression in the C2 subtype (Figure 4B).

### 3.5 Assocaition between SLC38A2 and glutathione metabolism

**FIGURE 5:**
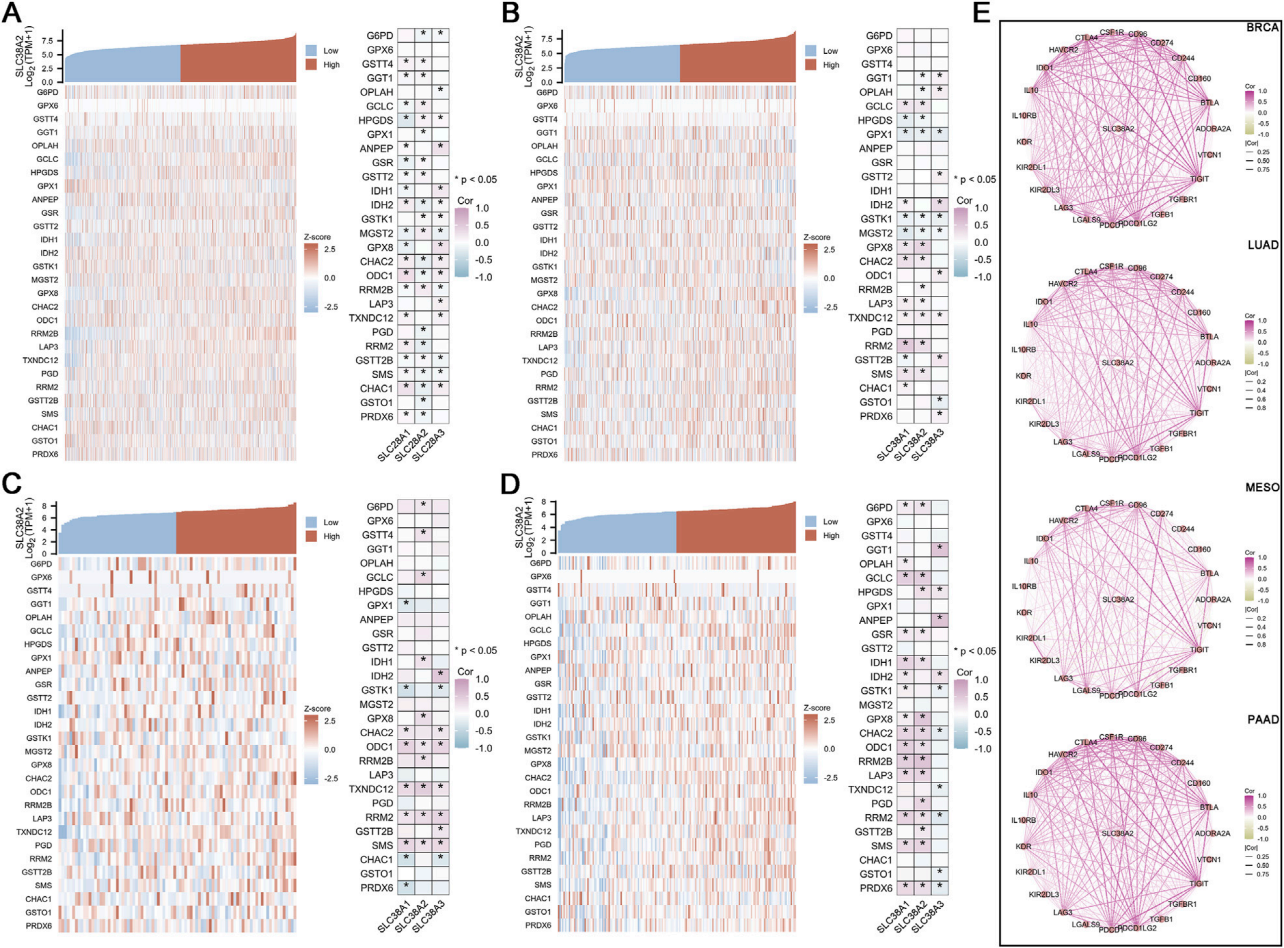
**(A)** Correlation between SLC38A2 and genes involved in glutathione metabolism in BRCA. **(B)** Correlation between SLC38A2 and genes involved in glutathione metabolism in LUAD. **(C)** Correlation between SLC38A2 and genes involved in glutathione metabolism in MESO. **(D)** Correlation between SLC38A2 and genes involved in glutathione metabolism in PAAD. **(E)** Correlation between SLC38A2 and immune checkpoint genes.

In BRCA, the expression of SLC38A2 is positively correlated with GSTT4, GGT1, GGLC, HPGDS, GSR, GSTT2, GSTK1, MGST2, RRM2B, and GSTT2B, while it is negatively correlated with G6PD, GPX1, IDH2, CHAC2, ODC1, PGD1, RRM2, SMS, CHAC1, GSTO1, and PRDX6 (Figure 5A). In LUAD, SLC38A2 shows positive correlations with GGLC, GPX8, CHAC2, RRM2B, LAP3, TXNDC12, RRM2, and SMS, and negative correlations with GGT1, OPLAH, HPGDS, GPX1, GSTK1, and MGST2 (Figure 5B). In MESO, SLC38A2 is positively correlated with G6PD, GSTT4, GCLC, IDH1, GPX8, ODC1, RRM2B, TXNDC12, RRM2, and SMS (Figure 5C). In PAAD, SLC38A2 exhibits positive correlations with G6PD, GCLC, HPGDS, GSR, IDH1, GPX8, CHAC2, ODC1, RRM2B, LAP3, PGD, RRM2, GSTT2B, SMS, and PRDX6 (Figure 5D).

**FIGURE 6:**
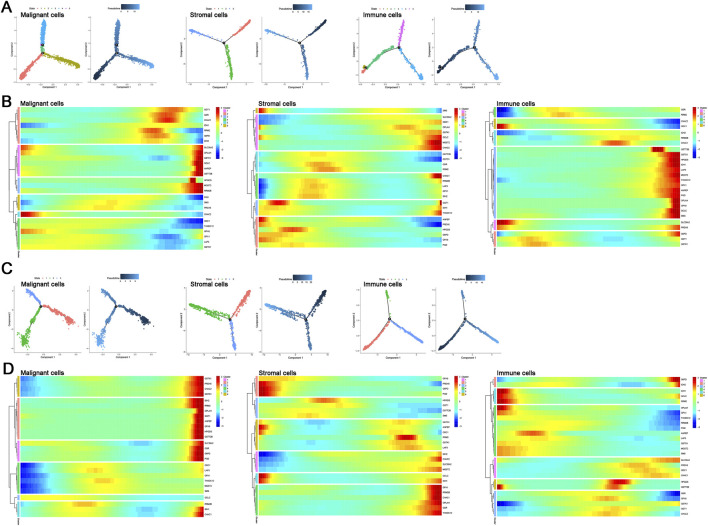
**(A)** Clustering of malignant cells, stromal cells, and immune cells in BRCA. **(B)** Temporal expression of SLC38A2 and glutamine metabolism-related genes in malignant, stromal, and immune cells in BRCA. **(C)** Clustering of malignant cells, stromal cells, and immune cells in PAAD. **(D)** Temporal expression of SLC38A2 and glutamine metabolism-related genes in malignant, stromal, and immune cells in PAAD.

In BRCA and PAAD, we categorized cells into three main types that include malignant cells, stromal cells, and immune cells. We analyzed the expression patterns of SLC38A2 and genes involved in glutathione metabolism within these cell types. The results indicate that SLC38A2 is highly expressed in the later stages across most cell subtypes, including malignant and stromal cells in BRCA, as well as malignant, stromal, and immune cells in PAAD. It is worth mentioning that SLC38A2 shows early enrichment in the immune cell subtype of BRCA (Figure 6).

### 3.6 Glutamine metabolism in COAD and LUAD

**FIGURE 7:**
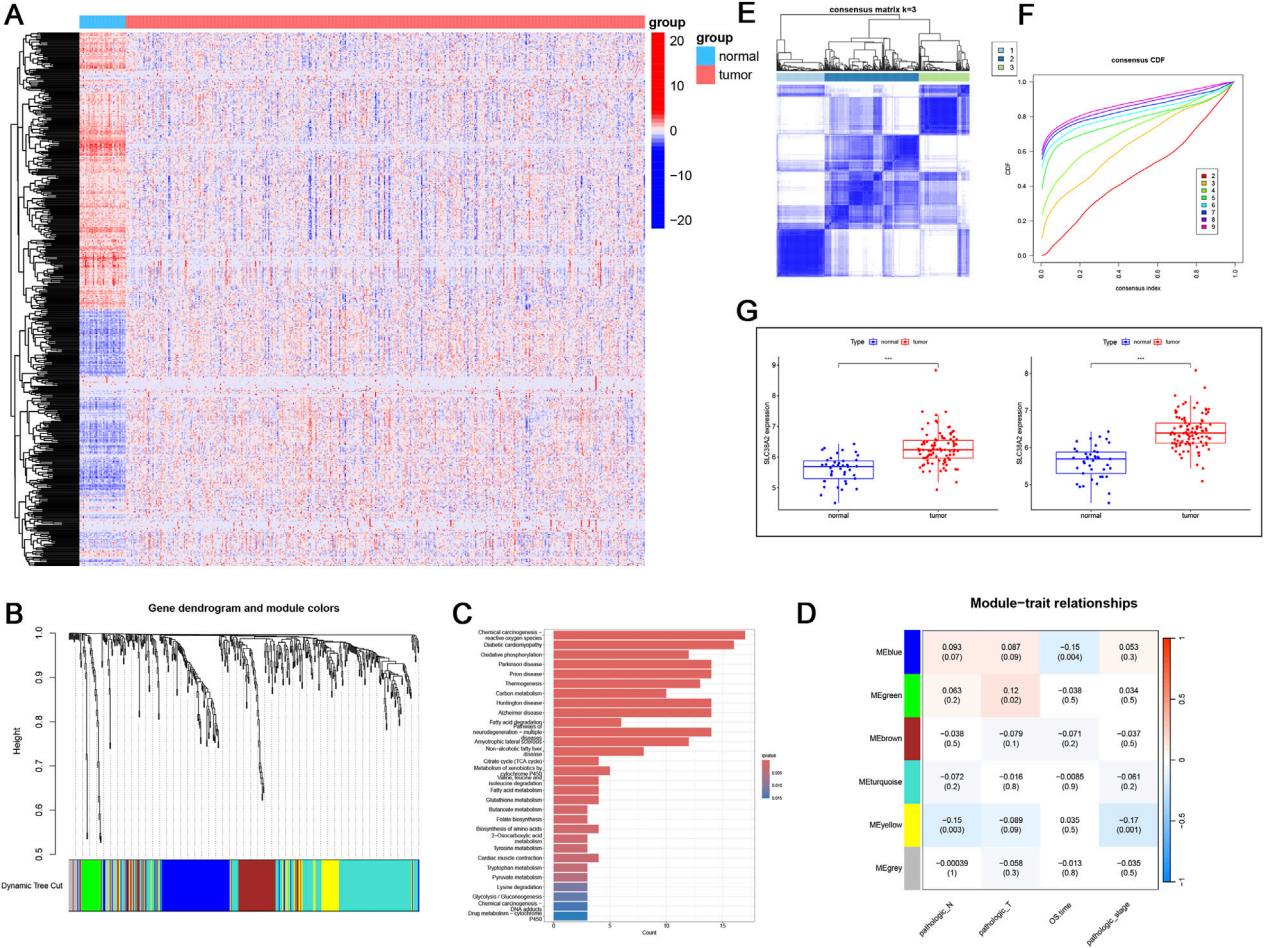
**(A)** Heatmap of the expression of 518 metabolic differential genes in COAD. **(B)** WGCNA clustering results based on metabolic differential genes. **(C)** KEGG enrichment analysis of genes in the yellow module. **(D)** Heatmap of the correlation between different clusters and clinical features. **(E)** Molecular subtype clustering of the yellow module (k=3). **(F)** Cumulative distribution function (CDF). **(G)** Expression of SLC38A2 in C1 and C3 clusters.

To further elucidate glutamine metabolism in pan-cancer and analyze its association with SLC38A2, we conducted further validation in COAD and LUAD. First, in COAD, we identified 518 differentially expressed genes out of 947 metabolic genes in the TCGA patient cohort, with 253 genes upregulated and 265 downregulated in tumors (Figure 7A). WGCNA results indicated that these genes could be well clustered into six distinct gene modules (Figure 7B). The yellow gene module was significantly associated with clinical indicators (pathologic_N = −0.15, pathologic_stage = −0.17) (Figure 7D). Functional enrichment analysis of the yellow module revealed enrichment in the glutamine metabolism pathway (Figure 7C). Next, we performed clustering analysis based on the expression levels of the 518 differentially expressed genes in the TCGA COAD cohort, grouping patients into three clusters (C1, C2, C3) (Figures 7E,F). We analyzed the expression levels of SLC38A2 in these patient subgroups and found higher expression in clusters C1 and C3 compared to normal samples (Figure 7G).

**FIGURE 8:**
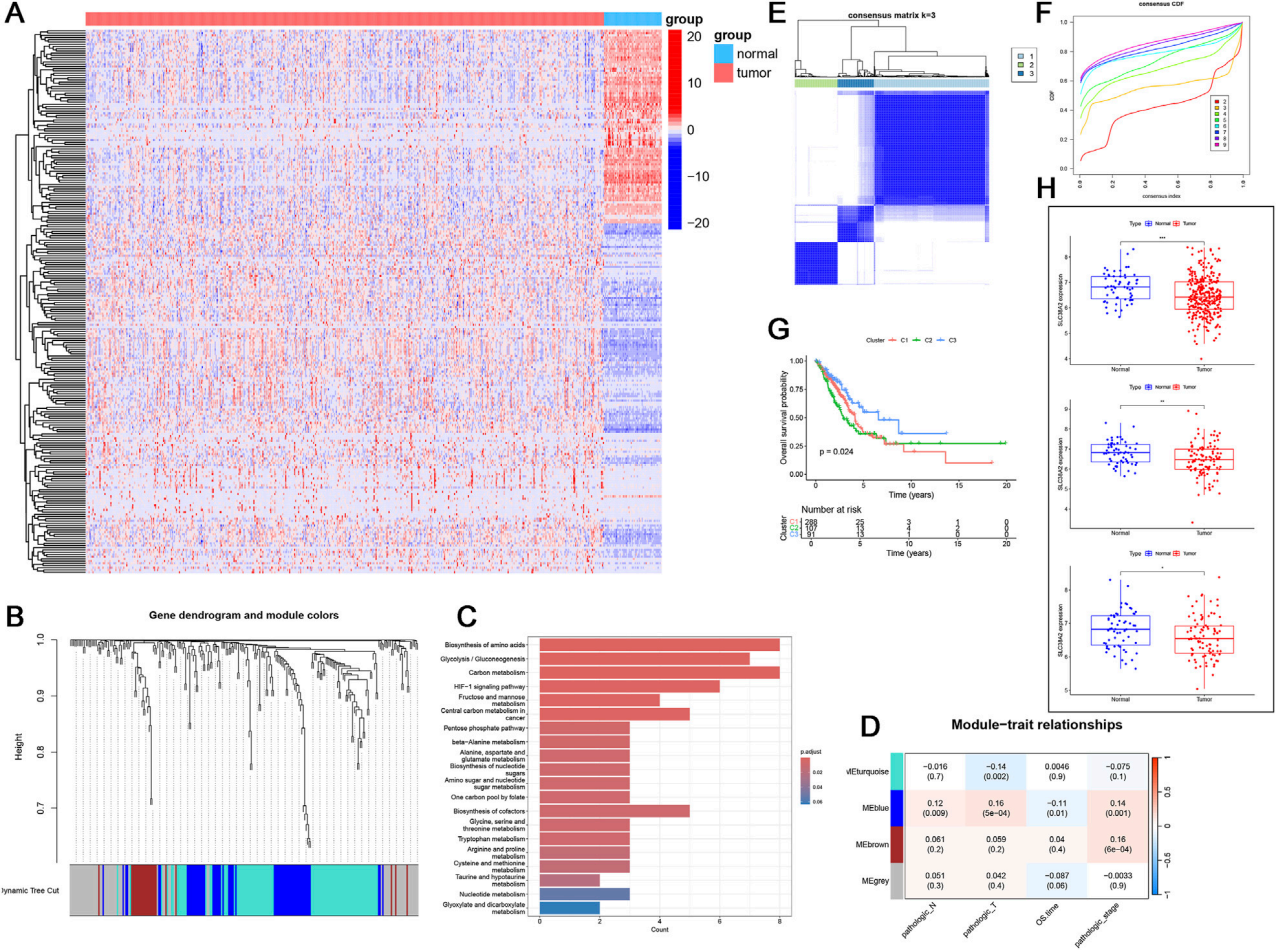
**(A)** Heatmap of the expression of 244 metabolic differential genes in LUAD. **(B)** WGCNA clustering results based on metabolic differential genes. **(C)** KEGG enrichment analysis of genes in the blue module. **(D)** Heatmap of the correlation between different clusters and clinical features. **(E)** Molecular subtype clustering of the blue module (k=3). **(F)** Cumulative distribution function (CDF). **(G)** Survival curves of patients in the three clusters. **(H)** Expression of SLC38A2 in C1, C2 and C3 clusters.

Similarly, in LUAD, we identified 244 differentially expressed metabolic genes and used WGCNA to cluster these genes into four gene modules (Figures 8A,B). The blue module was significantly associated with cancer prognosis (pathologic_N = 0.12, pathologic_T = 0.16, OS.time = −0.11, pathologic_stage = 0.14) (Figure 8D). Functional enrichment analysis of the blue module indicated that these genes are related to the glutamine metabolism pathway (Figure 8C). Further clustering results showed significant survival differences among the C1, C2, and C3 patient cohorts, with patients in the C3 group exhibiting normal survival rates (Figures 8E–G). Additionally, the expression levels of SLC38A2 in all three patient groups were lower than in normal samples (Figure 8H).

### 3.7 Assocaition between SLC38A2 and immune checkpoint

In BRCA, SLC38A2 expression is positively correlated with ADORA2A, CD160, CD274, CSF1R, HAVCR2, IL10, IL10RB, KDR, PDCD1LG2, TGFBR1, and VTCN1, and negatively correlated with IDO1, KIR2DL1, LAG3, LGALS9, and PDCD1. In LUAD, SLC38A2 expression is positively correlated with CD160, CD274, IL10, KDR, PDCD1LG2, and TGFBR1, and negatively correlated with LGALS9 and PDCD1. In MESO, the expression of SLC38A2 is positively correlated with CD160, IL10, KDR, TGFB1, and TGFBR1, and negatively correlated with LAG3 and LGALS9. And in PAAD, the expression of SLC38A2 is positively correlated with ADORA2A, BTLA, CD160, CD244, CD274, CD96, CSF1R, CTLA4, HAVCR2, IDO1, IL10, KDR, KIR2DL3, LAG3, PDCD1, PDCD1LG2, PVRL2, TGFB1, TGFBR1 and TIGIT (Figure 5E).

### 3.8 Signal pathways involving SLC38A2 in cancer

**FIGURE 9:**
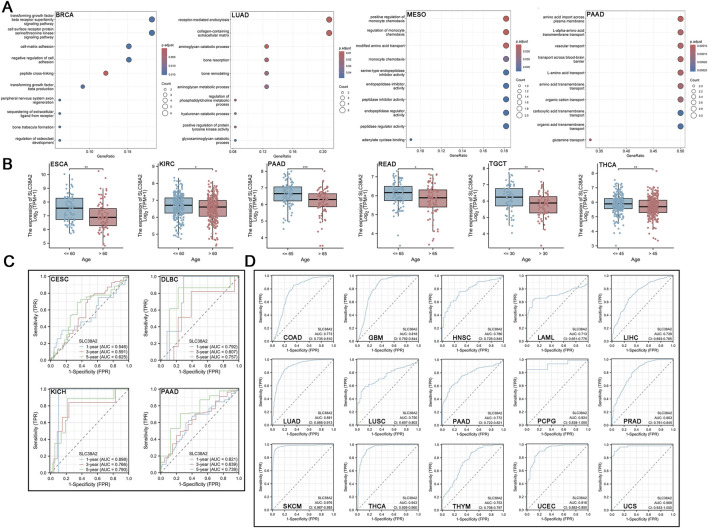
**(A)** Correlation of SLC38A2 with signaling pathways in BRCA, LUAD, MESO, and PAAD. **(B)** Expression of SLC38A2 in patients of different ages. **(C)** Time-dependent AUC curve using SLC38A2 as an evaluation metric, where an AUC value greater than 0.7 indicates predictive effectiveness. **(D)** Diagnostic ROC curve using SLC38A2 as an evaluation metric, where an ROC value greater than 0.7 indicates diagnostic effectiveness.

The GO enrichment analysis indicates that SLC38A2 is primarily associated with the following pathways in cancer. These include cell surface receptor protein serine/threonine kinase signaling pathway, transforming growth factor beta receptor superfamily signaling pathway, regulation of cell-matrix adhesion, regulation of bone formation and development, regulation of monocyte chemotaxis, and transmembrane transport of amino acids (Figure 9A).

### 3.9 SLC38A2-related cancer clinical features

First, we evaluated the correlation between SLC38A2 expression and patient age. We found that SLC38A2 expression was higher in younger patients, especially in six types of cancer: ESCA, KIRC, PAAD, READ, TGCT, and THCA (Figure 9B). Time-dependent AUC results indicate that SLC38A2 has a higher predictive value in three types of cancer that include DLBC (1-year AUC = 0.792, 5-year AUC = 0.757), KICH (1-year AUC = 0.898, 3-year AUC = 0.766, 5-year AUC = 0.790), and PAAD (5-year AUC = 0.739) (Figure 9C). Diagnostic ROC results show that SLC38A2 has diagnostic value in COAD, GBM, HNSC, LAML, LIHC, LUAD, LUSC, PAAD, PCPG, PRAD, SKCM, THCA, THYM, UCEC, and UCS (Figure 9D).

### 3.10 Assocaition between SLC38A2 and drug sensitivity

**FIGURE 10:**
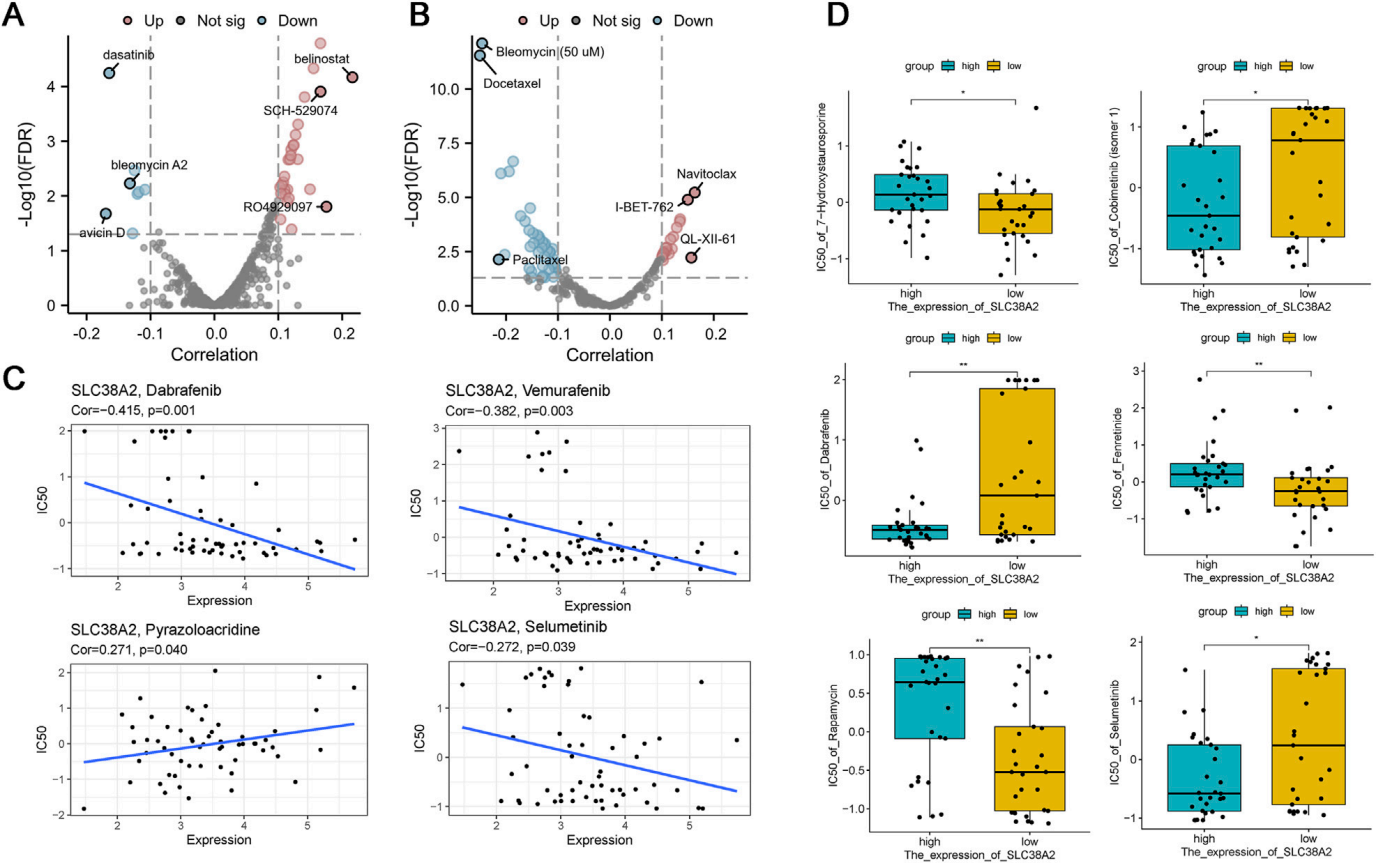
**(A)** Volcano plot showing the correlation between small molecule compound sensitivity and SLC38A2 expression in the CTRP database (Supplementary Material 2). **(B)** Volcano plot showing the correlation between small molecule compound sensitivity and SLC38A2 expression in the GDSC database (Supplementary Material 3). **(C)** Scatter plots showing the correlation between the sensitivity of four anticancer drugs and SLC38A2 expression. **(D)** Correlation between the sensitivity of six anticancer drugs and high/low SLC38A2 expression.

Results from the CTRP database indicate that the expression of SLC38A2 is significantly correlated with the sensitivity of 62 small-molecule compounds (Figure 10A), as well as the GDSC database results show a significant correlation between SLC38A2 expression and the sensitivity of 87 small-molecule compounds (*p* < 0.05) (Figure 10B). Additionally, SLC38A2 expression exhibits a moderate negative correlation with the sensitivity to Dabrafenib and Vemurafenib, a weak positive correlation with the sensitivity to Pyrazoloacridine, and a weak negative correlation with the sensitivity to Selumetinib (Figure 10C). Besides, we analyzed the relationship between the sensitivity of six chemotherapy drugs and the grouping based on SLC38A2 expression (Figure 10D).

## 4 Discussion

SLC38A2 (SNAT2), also known as Sodium-Coupled Neutral Amino Acid Transporter 2, is a transmembrane protein encoded by the locus at chromosome 12q13.11. It is widely expressed and regulated in mammalian cells, utilizing the sodium ion concentration gradient across the cell membrane to co-transport sodium ions and amino acid substrates into the cell (Franchi-Gazzola et al., 2006; Broer, 2014). Recent research has focused on the role of SLC38A2 in metabolic reprogramming and its connection to cancer progression. Abnormal metabolic patterns, often a hallmark of malignancies, are driven by uncontrolled division leading to abnormal proliferation. Metabolic reprogramming mechanisms such as aerobic glycolysis (the Warburg effect), hypoxia, and lactate accumulation are particularly notable. Glutamine, as an important alternative energy source, is deeply involved in the unique metabolic processes of tumor cells. Many tumors, such as glioblastoma, exhibit a strong affinity for glutamine, which supports tumor cell proliferation and survival (Huang et al., 2020). Additionally, glutamine “addiction” can lead to metabolic competition (Wise and Thompson, 2010), directly reducing uptake by immune cells, thereby threatening their integrity and function (Wilmore and Shabert, 1998). This has been confirmed in various cancers and *in vitro* models (Singer et al., 2018; Chang et al., 2015; Chen et al., 2023).

In the abnormal glutamine metabolism of tumors, the upregulated expression of selective transporters plays a crucial role, as it is essential for enhanced uptake by tumor cells (Bhutia et al., 2015). Several glutamine transporters have been confirmed to be widely upregulated in cancer and are associated with tumor invasion and metastasis, such as SLC1A5 (ASCT2), SLC7A5 (LAT1), and SLC6A14 (van Geldermalsen et al., 2016; Wu et al., 2018; Wang et al., 2013). The SLC38 family represents a new, not fully explored family of glutamine transporters, including system A [SLC38A1 (SNAT1) and SLC38A2] and system N [SLC38A3 (SNAT3) and SLC38A5 (SNAT5)]. Given its crucial role in the competitive uptake of glutamine by tumor cells, SLC38A2 is likely an effective molecular marker for reflecting tumor malignancy, immune suppression status, and clinical outcomes (Huang et al., 2020; Tambay et al., 2024). Several studies have demonstrated the significant role of SLC38A2 in the progression of certain tumors. For instance, research by Kai et al. indicates that overexpression of SLC38A2 is associated with the stemness of gastric cancer cells and is linked to tumor formation and upregulation of aldehyde dehydrogenase (ALDH) activity (Nie and Cai, 2022). Matteo et al.’s study suggests that the upregulation of SLC38A2 enhances glutamine-dependent resistance to oxidative stress and is correlated with worsened prognosis in triple-negative breast cancer (Morotti et al., 2021). However, the biological functions and prognostic implications of SLC38A2 in a broader range of tumors remain underexplored, posing a bottleneck for its targeting strategies and clinical applications.

To verify whether the expression of SLC38A2 is abnormal in cancer, we first conducted differential expression analysis across 33 common cancer types in the TCGA database. The analysis of non-paired samples revealed that the expression of SLC38A2 differs in 15 cancer types compared to their corresponding normal tissues, indicating altered expression in cancerous tissues. Furthermore, the immunohistochemistry slides further corroborated the differential expression results from the TCGA database.

After determining that SLC38A2 exhibits altered expression in cancer, we analyzed whether this gene affects cancer prognosis. Given the established biological functions of SLC38A2, we hypothesized that it is a risk factor for cancer prognosis. In our study, SLC38A2 was identified as a risk factor in BRCA, LUAD, MESO, and PAAD, where its high expression is associated with poor prognosis and shorter survival, aligning with other research findings (Morotti et al., 2021; Liu et al., 2022; Li et al., 2023a; Morotti et al., 2019). These results further support the link between the upregulation of SLC38A2 and the progression and increased malignancy of tumors, possibly achieved through its mediated enhancement of glutamine metabolism. Upregulation of SLC38A2 has been observed in triple-negative breast cancer and hormone-resistant breast cancer, and it is associated with resistance to oxidative stress and the development of drug resistance, ultimately leading to poor clinical outcomes (Morotti et al., 2021; Morotti et al., 2019). This corroborates our research findings. Interestingly, we found that SLC38A2 acts as a protective factor in certain cancers, particularly KIRC, where it is linked to favorable prognosis. A multi-platform data study showed widespread expression changes in the SLC family proteins in KIRC, with several SLC proteins (excluding SLC38A2) predicting improved OS and RFS (Kang et al., 2020). This result is consistent with another multi-omics-based bioinformatics study and supports our conclusions (Peng et al., 2022). However, the unique role of SLC38A2 in KIRC requires further investigation.

To further elucidate the role of SLC38A2 in tumor immunity, we conducted a comprehensive analysis of its correlation with the pan-cancer immune landscape. We found that SLC38A2 is positively correlated with stromal scores and negatively correlated with immune scores across various cancers, supporting the notion that SLC38A2 is a key player in tumor stroma remodeling and immune suppression. Research by Mengling et al. demonstrated that SLC38A2 is involved in glutamine crosstalk between TAMs and tumor cells in the Scissor_C1 cell subgroup of lung adenocarcinoma, accompanied by CAFs enrichment and epithelial-mesenchymal transition (EMT) phenotype (Li et al., 2023b).

It is worth northing that we found a significant positive correlation between SLC38A2 expression and the infiltration of T helper cells, Tcm cells, and Th2 cells. Th2 cells are a subset of CD4^+^T cells that promote tumor progression by secreting cytokines such as IL-10 and IL-13 to enhance angiogenesis and inhibit the cytotoxic effects of CD8^+^ T cells. In contrast, Tcm cells are primarily associated with immune memory. This evidence suggests that SLC38A2 regulates glutamine uptake in tumor and immune cells and plays a central role in adaptive T cell immunity. On one hand, the microenvironment acidification and amino acid competition driven by SLC38A2 may preferentially promote Th2 polarization. On the other hand, it might support the long-term survival and rapid response capabilities of Tcm cells, thereby enhancing immune memory effects. Hongling et al.’s study indicates that SLC38A2-mediated glutamine transport plays a crucial role in the generation and maintenance of memory T cells (Huang et al., 2021), which partially corroborates our findings. This dual effect reflects the complex role of SLC38A2 in tumor immune modulation. The overexpression of SLC38A2 enhances the nutritional tug-of-war between tumor cells and immune cells, inhibiting the activity of effector T cells (CD8^+^ T cells) while increasing the abundance of tumor-promoting Th2 cells. This ultimately results in an immunosuppressive state and poor clinical outcomes. In this process, memory immune cells may serve as a potential intervention target. Similarly, a single-cell RNA sequencing study indicated that the XBP1-SLC38A2 axis plays a significant role in T cell immune dysfunction in multiple myeloma, based on glutamine uptake impairment (Wan et al., 2023). Additionally, SLC38A2 shows a broad negative correlation with the infiltration of antigen-presenting cells such as DCs and NK cells. This reflects the critical role of SLC38A2-mediated glutamine competitive inhibition in blocking antigen presentation, leading to tumor immune suppression. Research by Hongsheng et al. demonstrated that exosomes derived from CAFs upregulate SLC38A2 expression on the surface of colorectal cancer cells, leading to NK cell exhaustion, thereby promoting CRC progression and metastasis (Fang et al., 2024). This conclusion is consistent with our findings.

To further elucidate the association between SLC38A2, the TME, and tumor-associated cells, we utilized single-cell sequencing data analysis (Li et al., 2023c; Ding et al., 2024) to evaluate the expression patterns of SLC38A2 across 12 types of cancer. At the single-cell level, SLC38A2 is primarily expressed in stromal cells. Abnormal extracellular matrix is a key factor in promoting tumor progression and mediating drug resistance and immune evasion. Collagen deposition and tumor stiffening caused by over cancer-associated fibroblasts (CAFs) proliferation exacerbate hypoxia, facilitating metabolic reprogramming of tumor cells and increasing glutamine uptake through crosstalk (Bertero et al., 2019). The overexpression of transporter proteins by stromal cells further depletes glutamine in TME, potentially leading to immunosuppression. Xiao et al.’s study indicates that CAFs increase the expression of the glutamine transporter SLC1A5 and significantly enhance the synthesis of nitrogen-containing compounds to fuel tumor cells (Li et al., 2021b), supporting our findings. Additionally, breast cancer cells co-cultured with CAFs show increased metastatic and migratory capabilities, linked to microRNA-mediated glutamine metabolic reprogramming, which may lead to poor clinical outcomes (Yan et al., 2018). And research by Tongyan et al. showed that in LUAD, CAFs mediate the upregulation of SLC38A2 through the lncRNA LINC01614, interacting with tumor cells to result in poor clinical outcomes (Liu et al., 2022). Recent studies on the role of SLC38A2 in tumors and targeted therapies have focused on BRCA (Morotti et al., 2019; Gauthier-Coles et al., 2022; Broer et al., 2019). We observed significant differences in the timing of SLC38A2 expression across different cell subpopulations in BRCA. In malignant and stromal cell subgroups, SLC38A2 is primarily expressed at the late stages of differentiation. Conversely, in immune cells, it is mainly expressed at the early stages of differentiation. This indicates that SLC38A2 becomes irreversibly enriched in the “unfavorable” cell subgroups during the late stages of tumor progression, ultimately leading to immune cell exhaustion due to glutamine deprivation. Additionally, the WGCNA results further demonstrate the role of the glutamine metabolic pathway in certain malignant tumors and emphasize its inseparable connection with SLC38A2.

Finally, we validated whether SLC38A2 could serve as an effective prognostic and diagnostic biomarker. We found that SLC38A2 is expressed at higher levels in younger patients with certain cancers, potentially indicating increased risk. Additionally, diagnostic ROC and time-dependent AUC results demonstrate that SLC38A2 is indeed a reliable diagnostic marker in some cancers, further supporting its clinical application potential.

Although many studies have confirmed that blocking glutamine metabolism pathways significantly inhibits tumor progression, research and targeted drugs specifically for SLC38A2 remain limited (Wang et al., 2024; Li et al., 2023d; Jin et al., 2023). Our study further supports the important prognostic and diagnostic value of SLC38A2 and analyzes its biological role in various tumors. These results suggest that strategies block the overexpression of SLC38A2 in tumor or stromal cells to alleviate glutamine competition in TME, or to enhance the glutamine uptake capacity of specific immune cells, could be effective. This warrants further in-depth research.

## 5 Conclusion

This study further confirms the role of the key transporter SLC38A2 in glutamine metabolism across various cancers, encompassing clinical aspects, the tumor microenvironment, immunity, and drug sensitivity. SLC38A2 is closely linked to glutamine metabolic mechanisms and serves as an effective prognostic and diagnostic marker associated with poor clinical outcomes. Its unique association with inhibitory immune cells and stromal cells makes it a potential target for intervening in the tumor microenvironment, warranting further in-depth research.

## Data Availability

Publicly available datasets were analyzed in this study. This data can be found here: https://www.ncbi.nlm.nih.gov/.
