# Supplementary material for: Panoramic analysis of the biological function and clinical value of SLC38A2 in human cancers: a study based on pan-cancer and single-cell analysis
Source: Front Genet. 2025 Sep 17;16:1658299. doi: 10.3389/fgene.2025.1658299 (PMC12483874; doi:10.3389/fgene.2025.1658299)
Supplement: Supplementary file 3 [file Table1.docx]

**Supplementary Material 1**

Multivariate Cox Regression Analysis of SLC38A2 in Pan-Cancer. Using Overall Survival (OS) as the outcome variable, categorized by median.

**1. ACC**

| Characteristics | Total(N) | Univariate analysis | |  | Multivariate analysis | |
| --- | --- | --- | --- | --- | --- | --- |
|  |  | Hazard ratio (95% CI) | P value |  | Hazard ratio (95% CI) | P value |
| Pathologic T stage | 77 |  |  |  |  |  |
| T1 | 9 | Reference |  |  | Reference |  |
| T2 | 42 | 2.444 (0.305 - 19.596) | 0.400 |  | 1.989 (0.243 - 16.247) | 0.521 |
| T3 | 8 | 12.439 (1.300 - 118.988) | **0.029** |  | 13.699 (1.324 - 141.761) | **0.028** |
| T4 | 18 | 30.506 (3.595 - 258.857) | **0.002** |  | 22.976 (2.369 - 222.870) | **0.007** |
| Pathologic N stage | 77 |  |  |  |  |  |
| N0 | 68 | Reference |  |  | Reference |  |
| N1 | 9 | 2.038 (0.769 - 5.400) | 0.152 |  | 1.638 (0.531 - 5.049) | 0.390 |
| Clinical M stage | 77 |  |  |  |  |  |
| M0 | 62 | Reference |  |  | Reference |  |
| M1 | 15 | 6.150 (2.710 - 13.959) | **< 0.001** |  | 1.252 (0.430 - 3.647) | 0.680 |
| Gender | 79 |  |  |  |  |  |
| Female | 48 | Reference |  |  | Reference |  |
| Male | 31 | 1.001 (0.469 - 2.137) | 0.999 |  | 0.739 (0.321 - 1.701) | 0.478 |
| Age | 79 |  |  |  |  |  |
| <= 50 | 41 | Reference |  |  | Reference |  |
| > 50 | 38 | 1.799 (0.846 - 3.824) | 0.127 |  | 2.147 (0.906 - 5.085) | 0.083 |
| SLC38A2 | 79 |  |  |  |  |  |
| Low | 39 | Reference |  |  | Reference |  |
| High | 40 | 1.346 (0.639 - 2.836) | 0.435 |  | 1.143 (0.493 - 2.651) | 0.755 |

**2. BLCA**

| Characteristics | Total(N) | Univariate analysis | |  | Multivariate analysis | |
| --- | --- | --- | --- | --- | --- | --- |
|  |  | Hazard ratio (95% CI) | P value |  | Hazard ratio (95% CI) | P value |
| Pathologic T stage | 377 |  |  |  |  |  |
| T1&T2 | 123 | Reference |  |  | Reference |  |
| T3 | 195 | 1.970 (1.339 - 2.899) | **< 0.001** |  | 1.817 (0.935 - 3.529) | 0.078 |
| T4 | 59 | 2.987 (1.860 - 4.797) | **< 0.001** |  | 2.476 (1.100 - 5.571) | **0.028** |
| Pathologic N stage | 367 |  |  |  |  |  |
| N0 | 238 | Reference |  |  | Reference |  |
| N1 | 46 | 1.844 (1.189 - 2.857) | **0.006** |  | 1.781 (0.920 - 3.447) | 0.087 |
| N2&N3 | 83 | 2.527 (1.786 - 3.576) | **< 0.001** |  | 1.962 (1.076 - 3.574) | **0.028** |
| Pathologic M stage | 212 |  |  |  |  |  |
| M0 | 201 | Reference |  |  | Reference |  |
| M1 | 11 | 3.112 (1.491 - 6.493) | **0.002** |  | 1.533 (0.565 - 4.165) | 0.402 |
| Gender | 411 |  |  |  |  |  |
| Female | 108 | Reference |  |  | Reference |  |
| Male | 303 | 0.868 (0.629 - 1.198) | 0.390 |  | 0.614 (0.368 - 1.027) | 0.063 |
| Age | 411 |  |  |  |  |  |
| <= 70 | 231 | Reference |  |  | Reference |  |
| > 70 | 180 | 1.424 (1.064 - 1.906) | **0.018** |  | 1.187 (0.740 - 1.902) | 0.477 |
| SLC38A2 | 411 |  |  |  |  |  |
| Low | 205 | Reference |  |  | Reference |  |
| High | 206 | 0.999 (0.746 - 1.338) | 0.995 |  | 1.506 (0.928 - 2.443) | 0.097 |

**3. BRCA**

| Characteristics | Total(N) | Univariate analysis | |  | Multivariate analysis | |
| --- | --- | --- | --- | --- | --- | --- |
|  |  | Hazard ratio (95% CI) | P value |  | Hazard ratio (95% CI) | P value |
| Pathologic T stage | 1,083 |  |  |  |  |  |
| T1 | 277 | Reference |  |  | Reference |  |
| T2 | 631 | 1.334 (0.889 - 2.003) | 0.164 |  | 1.255 (0.791 - 1.989) | 0.335 |
| T3&T4 | 175 | 1.931 (1.208 - 3.088) | **0.006** |  | 1.392 (0.790 - 2.453) | 0.253 |
| Pathologic N stage | 1,067 |  |  |  |  |  |
| N0 | 516 | Reference |  |  | Reference |  |
| N1 | 358 | 1.947 (1.322 - 2.865) | **< 0.001** |  | 1.862 (1.225 - 2.829) | **0.004** |
| N2 | 116 | 2.522 (1.484 - 4.287) | **< 0.001** |  | 2.568 (1.436 - 4.593) | **0.001** |
| N3 | 77 | 4.191 (2.318 - 7.580) | **< 0.001** |  | 3.356 (1.613 - 6.985) | **0.001** |
| Pathologic M stage | 925 |  |  |  |  |  |
| M0 | 905 | Reference |  |  | Reference |  |
| M1 | 20 | 4.266 (2.474 - 7.354) | **< 0.001** |  | 1.938 (0.997 - 3.769) | 0.051 |
| Age | 1,086 |  |  |  |  |  |
| <= 60 | 603 | Reference |  |  | Reference |  |
| > 60 | 483 | 2.024 (1.468 - 2.790) | **< 0.001** |  | 2.295 (1.597 - 3.298) | **< 0.001** |
| SLC38A2 | 1,086 |  |  |  |  |  |
| Low | 543 | Reference |  |  | Reference |  |
| High | 543 | 1.370 (0.992 - 1.890) | 0.056 |  | 1.473 (1.028 - 2.110) | **0.035** |

**4. CESC**

| Characteristics | Total(N) | Univariate analysis | |  | Multivariate analysis | |
| --- | --- | --- | --- | --- | --- | --- |
|  |  | Hazard ratio (95% CI) | P value |  | Hazard ratio (95% CI) | P value |
| Pathologic T stage | 243 |  |  |  |  |  |
| T1 | 140 | Reference |  |  | Reference |  |
| T2 | 72 | 1.140 (0.557 - 2.333) | 0.720 |  | 0.409 (0.134 - 1.253) | 0.118 |
| T3&T4 | 31 | 4.019 (2.072 - 7.797) | **< 0.001** |  | 3.343 (1.183 - 9.445) | **0.023** |
| Pathologic N stage | 195 |  |  |  |  |  |
| N0 | 134 | Reference |  |  | Reference |  |
| N1 | 61 | 2.844 (1.446 - 5.593) | **0.002** |  | 3.019 (1.475 - 6.179) | **0.002** |
| Pathologic M stage | 256 |  |  |  |  |  |
| M0 | 116 | Reference |  |  | Reference |  |
| M1 | 11 | 3.651 (1.226 - 10.872) | **0.020** |  | 0.000 (0.000 - Inf) | 0.997 |
| MX | 129 | 1.973 (1.112 - 3.501) | **0.020** |  | 1.535 (0.743 - 3.172) | 0.247 |
| Age | 306 |  |  |  |  |  |
| <= 50 | 188 | Reference |  |  | Reference |  |
| > 50 | 118 | 1.289 (0.810 - 2.050) | 0.284 |  | 0.961 (0.441 - 2.095) | 0.921 |
| SLC38A2 | 306 |  |  |  |  |  |
| Low | 153 | Reference |  |  | Reference |  |
| High | 153 | 1.572 (0.982 - 2.518) | 0.059 |  | 1.839 (0.883 - 3.832) | 0.104 |

**5. CHOL**

| Characteristics | Total(N) | Univariate analysis | |  | Multivariate analysis | |
| --- | --- | --- | --- | --- | --- | --- |
|  |  | Hazard ratio (95% CI) | P value |  | Hazard ratio (95% CI) | P value |
| Pathologic T stage | 35 |  |  |  |  |  |
| T1 | 18 | Reference |  |  | Reference |  |
| T2 | 12 | 2.370 (0.853 - 6.585) | 0.098 |  | 2.217 (0.469 - 10.474) | 0.315 |
| T3&T4 | 5 | 0.892 (0.184 - 4.312) | 0.887 |  | 0.517 (0.037 - 7.258) | 0.624 |
| Pathologic N stage | 30 |  |  |  |  |  |
| N0 | 25 | Reference |  |  | Reference |  |
| N1 | 5 | 2.147 (0.565 - 8.154) | 0.262 |  | 2.242 (0.296 - 17.010) | 0.435 |
| Pathologic M stage | 32 |  |  |  |  |  |
| M0 | 27 | Reference |  |  | Reference |  |
| M1 | 5 | 1.531 (0.428 - 5.475) | 0.513 |  | 0.973 (0.064 - 14.778) | 0.984 |
| Gender | 35 |  |  |  |  |  |
| Female | 19 | Reference |  |  | Reference |  |
| Male | 16 | 1.279 (0.503 - 3.255) | 0.605 |  | 1.155 (0.277 - 4.819) | 0.843 |
| Age | 35 |  |  |  |  |  |
| <= 65 | 17 | Reference |  |  | Reference |  |
| > 65 | 18 | 1.461 (0.570 - 3.747) | 0.430 |  | 2.847 (0.778 - 10.414) | 0.114 |
| SLC38A2 | 35 |  |  |  |  |  |
| Low | 17 | Reference |  |  | Reference |  |
| High | 18 | 1.298 (0.511 - 3.298) | 0.583 |  | 1.227 (0.368 - 4.089) | 0.739 |

**6. COAD**

| Characteristics | Total(N) | Univariate analysis | |  | Multivariate analysis | |
| --- | --- | --- | --- | --- | --- | --- |
|  |  | Hazard ratio (95% CI) | P value |  | Hazard ratio (95% CI) | P value |
| Pathologic T stage | 476 |  |  |  |  |  |
| T1&T2 | 94 | Reference |  |  | Reference |  |
| T3 | 322 | 2.576 (1.183 - 5.612) | **0.017** |  | 3.371 (1.030 - 11.032) | **0.044** |
| T4 | 60 | 7.021 (2.993 - 16.473) | **< 0.001** |  | 6.943 (1.943 - 24.812) | **0.003** |
| Pathologic N stage | 477 |  |  |  |  |  |
| N0 | 283 | Reference |  |  | Reference |  |
| N1 | 108 | 1.681 (1.019 - 2.771) | **0.042** |  | 1.161 (0.634 - 2.126) | 0.629 |
| N2 | 86 | 4.051 (2.593 - 6.329) | **< 0.001** |  | 2.402 (1.347 - 4.283) | **0.003** |
| Pathologic M stage | 414 |  |  |  |  |  |
| M0 | 348 | Reference |  |  | Reference |  |
| M1 | 66 | 4.193 (2.683 - 6.554) | **< 0.001** |  | 2.510 (1.438 - 4.382) | **0.001** |
| Gender | 477 |  |  |  |  |  |
| Female | 226 | Reference |  |  | Reference |  |
| Male | 251 | 1.101 (0.746 - 1.625) | 0.627 |  | 0.801 (0.519 - 1.236) | 0.315 |
| Age | 477 |  |  |  |  |  |
| <= 65 | 194 | Reference |  |  | Reference |  |
| > 65 | 283 | 1.610 (1.052 - 2.463) | **0.028** |  | 2.013 (1.233 - 3.288) | **0.005** |
| SLC38A2 | 477 |  |  |  |  |  |
| Low | 238 | Reference |  |  | Reference |  |
| High | 239 | 0.832 (0.564 - 1.226) | 0.352 |  | 0.666 (0.433 - 1.024) | **0.044** |

**7. DLBC**

| Characteristics | Total(N) | Univariate analysis | |  | Multivariate analysis | |
| --- | --- | --- | --- | --- | --- | --- |
|  |  | Hazard ratio (95% CI) | P value |  | Hazard ratio (95% CI) | P value |
| Clinical stage | 42 |  |  |  |  |  |
| Stage I | 8 | Reference |  |  | Reference |  |
| Stage II | 17 | 1.053 (0.093 - 11.943) | 0.967 |  | 0.192 (0.008 - 4.433) | 0.303 |
| Stage III | 5 | 1.042 (0.061 - 17.896) | 0.978 |  | 0.565 (0.017 - 18.433) | 0.748 |
| Stage IV | 12 | 2.703 (0.261 - 28.021) | 0.405 |  | 0.283 (0.016 - 5.097) | 0.392 |
| Primary therapy outcome | 46 |  |  |  |  |  |
| PD | 5 | Reference |  |  | Reference |  |
| SD | 3 | 0.350 (0.036 - 3.414) | 0.366 |  | 1.045 (0.052 - 20.987) | 0.977 |
| PR | 3 | 0.000 (0.000 - Inf) | 0.999 |  | 0.000 (0.000 - Inf) | 0.999 |
| CR | 35 | 0.069 (0.013 - 0.357) | **0.001** |  | 0.028 (0.002 - 0.409) | **0.009** |
| Gender | 48 |  |  |  |  |  |
| Female | 26 | Reference |  |  | Reference |  |
| Male | 22 | 1.039 (0.250 - 4.324) | 0.958 |  | 0.487 (0.076 - 3.106) | 0.447 |
| Age | 48 |  |  |  |  |  |
| <= 60 | 27 | Reference |  |  | Reference |  |
| > 60 | 21 | 1.666 (0.416 - 6.673) | 0.471 |  | 1.596 (0.151 - 16.869) | 0.698 |
| SLC38A2 | 48 |  |  |  |  |  |
| Low | 24 | Reference |  |  | Reference |  |
| High | 24 | 4.045 (0.785 - 20.846) | 0.095 |  | 13.105 (1.115 - 153.985) | **0.041** |

**8. ESCA**

| Characteristics | Total(N) | Univariate analysis | |  | Multivariate analysis | |
| --- | --- | --- | --- | --- | --- | --- |
|  |  | Hazard ratio (95% CI) | P value |  | Hazard ratio (95% CI) | P value |
| Pathologic T stage | 145 |  |  |  |  |  |
| T1 | 27 | Reference |  |  | Reference |  |
| T2 | 37 | 0.868 (0.388 - 1.940) | 0.729 |  | 1.246 (0.486 - 3.196) | 0.647 |
| T3&T4 | 81 | 1.211 (0.599 - 2.450) | 0.594 |  | 1.546 (0.681 - 3.510) | 0.298 |
| Pathologic N stage | 144 |  |  |  |  |  |
| N0 | 66 | Reference |  |  | Reference |  |
| N1 | 63 | 2.853 (1.515 - 5.373) | **0.001** |  | 2.437 (1.166 - 5.094) | **0.018** |
| N2&N3 | 15 | 3.602 (1.483 - 8.745) | **0.005** |  | 4.262 (1.456 - 12.479) | **0.008** |
| Pathologic M stage | 129 |  |  |  |  |  |
| M0 | 121 | Reference |  |  | Reference |  |
| M1 | 8 | 5.075 (2.312 - 11.136) | **< 0.001** |  | 4.444 (1.795 - 10.997) | **0.001** |
| Gender | 163 |  |  |  |  |  |
| Female | 23 | Reference |  |  | Reference |  |
| Male | 140 | 2.338 (0.935 - 5.846) | 0.069 |  | 2.076 (0.607 - 7.103) | 0.244 |
| Age | 163 |  |  |  |  |  |
| <= 60 | 83 | Reference |  |  | Reference |  |
| > 60 | 80 | 0.858 (0.525 - 1.402) | 0.541 |  | 1.005 (0.538 - 1.876) | 0.987 |
| SLC38A2 | 163 |  |  |  |  |  |
| Low | 81 | Reference |  |  | Reference |  |
| High | 82 | 1.009 (0.612 - 1.662) | 0.974 |  | 0.868 (0.466 - 1.619) | 0.657 |

**9. GBM**

| Characteristics | Total(N) | Univariate analysis | |  | Multivariate analysis | |
| --- | --- | --- | --- | --- | --- | --- |
|  |  | Hazard ratio (95% CI) | P value |  | Hazard ratio (95% CI) | P value |
| IDH status | 161 |  |  |  |  |  |
| WT | 149 | Reference |  |  | Reference |  |
| Mut | 12 | 0.301 (0.138 - 0.654) | **0.002** |  | 0.311 (0.138 - 0.700) | **0.005** |
| Gender | 168 |  |  |  |  |  |
| Female | 59 | Reference |  |  | Reference |  |
| Male | 109 | 1.026 (0.719 - 1.466) | 0.887 |  | 1.099 (0.755 - 1.600) | 0.623 |
| Age | 168 |  |  |  |  |  |
| <= 60 | 87 | Reference |  |  | Reference |  |
| > 60 | 81 | 1.365 (0.973 - 1.915) | 0.072 |  | 1.086 (0.759 - 1.554) | 0.651 |
| SLC38A2 | 168 |  |  |  |  |  |
| Low | 84 | Reference |  |  | Reference |  |
| High | 84 | 0.809 (0.575 - 1.137) | 0.222 |  | 0.844 (0.591 - 1.206) | 0.352 |

**10. HNSC**

| Characteristics | Total(N) | Univariate analysis | |  | Multivariate analysis | |
| --- | --- | --- | --- | --- | --- | --- |
|  |  | Hazard ratio (95% CI) | P value |  | Hazard ratio (95% CI) | P value |
| Pathologic T stage | 447 |  |  |  |  |  |
| T1 | 45 | Reference |  |  | Reference |  |
| T2 | 134 | 1.341 (0.694 - 2.590) | 0.382 |  | 0.604 (0.190 - 1.921) | 0.393 |
| T3 | 96 | 2.592 (1.349 - 4.984) | **0.004** |  | 1.714 (0.548 - 5.358) | 0.354 |
| T4 | 172 | 2.347 (1.250 - 4.407) | **0.008** |  | 1.890 (0.695 - 5.138) | 0.212 |
| Pathologic N stage | 410 |  |  |  |  |  |
| N0 | 170 | Reference |  |  | Reference |  |
| N1 | 66 | 0.957 (0.563 - 1.624) | 0.870 |  | 1.066 (0.443 - 2.565) | 0.886 |
| N2&N3 | 174 | 2.269 (1.617 - 3.183) | **< 0.001** |  | 2.820 (1.576 - 5.045) | **< 0.001** |
| Pathologic M stage | 188 |  |  |  |  |  |
| M0 | 187 | Reference |  |  | Reference |  |
| M1 | 1 | 22.631 (2.830 - 180.948) | **0.003** |  | 24.101 (2.265 - 256.470) | **0.008** |
| Gender | 503 |  |  |  |  |  |
| Female | 134 | Reference |  |  | Reference |  |
| Male | 369 | 0.760 (0.571 - 1.012) | 0.061 |  | 0.677 (0.378 - 1.212) | 0.189 |
| Age | 503 |  |  |  |  |  |
| <= 60 | 247 | Reference |  |  | Reference |  |
| > 60 | 256 | 1.262 (0.964 - 1.653) | 0.090 |  | 0.949 (0.546 - 1.650) | 0.853 |
| SLC38A2 | 503 |  |  |  |  |  |
| Low | 251 | Reference |  |  | Reference |  |
| High | 252 | 1.235 (0.945 - 1.614) | 0.123 |  | 1.303 (0.777 - 2.185) | 0.316 |

**11. KICH**

| Characteristics | Total(N) | Univariate analysis | |  | Multivariate analysis | |
| --- | --- | --- | --- | --- | --- | --- |
|  |  | Hazard ratio (95% CI) | P value |  | Hazard ratio (95% CI) | P value |
| Pathologic T stage | 64 |  |  |  |  |  |
| T1 | 19 | Reference |  |  | Reference |  |
| T2 | 25 | 251392806.6641 (0.000 - Inf) | 0.999 |  | 0.000 (0.000 - Inf) | 1.000 |
| T3&T4 | 20 | 1400423816.8264 (0.000 - Inf) | 0.999 |  | 1.453 (0.000 - Inf) | 1.000 |
| Pathologic N stage | 43 |  |  |  |  |  |
| N0 | 38 | Reference |  |  | Reference |  |
| N1&N2 | 5 | 30240971662.2690 (0.000 - Inf) | 0.999 |  | 98720715165991508295811072.0000 (0.000 - Inf) | 1.000 |
| Pathologic M stage | 36 |  |  |  |  |  |
| M0 | 34 | Reference |  |  | Reference |  |
| M1 | 2 | 16.493 (1.495 - 181.921) | **0.022** |  | 0.000 (0.000 - Inf) | 0.999 |
| Gender | 64 |  |  |  |  |  |
| Female | 26 | Reference |  |  | Reference |  |
| Male | 38 | 1.528 (0.381 - 6.125) | 0.550 |  | 61259850513118.1016 (0.000 - Inf) | 0.999 |
| Age | 64 |  |  |  |  |  |
| <= 50 | 33 | Reference |  |  | Reference |  |
| > 50 | 31 | 3.847 (0.797 - 18.561) | 0.093 |  | 0.000 (0.000 - Inf) | 1.000 |
| SLC38A2 | 64 |  |  |  |  |  |
| Low | 32 | Reference |  |  | Reference |  |
| High | 32 | 9.416 (1.176 - 75.419) | **0.035** |  | 18191201056842.3984 (0.000 - Inf) | 0.999 |

**12. KIRC**

| Characteristics | Total(N) | Univariate analysis | |  | Multivariate analysis | |
| --- | --- | --- | --- | --- | --- | --- |
|  |  | Hazard ratio (95% CI) | P value |  | Hazard ratio (95% CI) | P value |
| Pathologic T stage | 541 |  |  |  |  |  |
| T1 | 279 | Reference |  |  | Reference |  |
| T2 | 71 | 1.490 (0.895 - 2.481) | 0.125 |  | 1.140 (0.666 - 1.950) | 0.632 |
| T3&T4 | 191 | 3.555 (2.536 - 4.982) | **< 0.001** |  | 2.070 (1.395 - 3.071) | **< 0.001** |
| Pathologic M stage | 508 |  |  |  |  |  |
| M0 | 429 | Reference |  |  | Reference |  |
| M1 | 79 | 4.401 (3.226 - 6.002) | **< 0.001** |  | 3.109 (2.168 - 4.457) | **< 0.001** |
| Gender | 541 |  |  |  |  |  |
| Female | 187 | Reference |  |  | Reference |  |
| Male | 354 | 0.924 (0.679 - 1.257) | 0.613 |  | 0.865 (0.631 - 1.185) | 0.366 |
| Age | 541 |  |  |  |  |  |
| <= 60 | 269 | Reference |  |  | Reference |  |
| > 60 | 272 | 1.791 (1.319 - 2.432) | **< 0.001** |  | 1.717 (1.256 - 2.347) | **< 0.001** |
| SLC38A2 | 541 |  |  |  |  |  |
| Low | 270 | Reference |  |  | Reference |  |
| High | 271 | 0.750 (0.555 - 1.012) | 0.060 |  | 0.815 (0.600 - 1.107) | 0.190 |

**13. KIRP**

| Characteristics | Total(N) | Univariate analysis | |  | Multivariate analysis | |
| --- | --- | --- | --- | --- | --- | --- |
|  |  | Hazard ratio (95% CI) | P value |  | Hazard ratio (95% CI) | P value |
| Pathologic T stage | 288 |  |  |  |  |  |
| T1 | 192 | Reference |  |  | Reference |  |
| T2 | 34 | 2.959 (1.224 - 7.157) | **0.016** |  | 0.982 (0.205 - 4.696) | 0.982 |
| T3&T4 | 62 | 6.504 (3.254 - 12.999) | **< 0.001** |  | 1.042 (0.306 - 3.546) | 0.947 |
| Pathologic N stage | 78 |  |  |  |  |  |
| N0 | 50 | Reference |  |  | Reference |  |
| N1 | 24 | 5.030 (2.016 - 12.550) | **< 0.001** |  | 2.335 (0.656 - 8.320) | 0.191 |
| N2 | 4 | 6.300 (1.619 - 24.525) | **0.008** |  | 0.000 (0.000 - Inf) | 0.996 |
| Pathologic M stage | 104 |  |  |  |  |  |
| M0 | 95 | Reference |  |  | Reference |  |
| M1 | 9 | 114.966 (22.481 - 587.925) | **< 0.001** |  | 42.348 (4.955 - 361.918) | **< 0.001** |
| Gender | 290 |  |  |  |  |  |
| Female | 77 | Reference |  |  | Reference |  |
| Male | 213 | 0.630 (0.327 - 1.213) | 0.167 |  | 0.560 (0.141 - 2.230) | 0.411 |
| Age | 288 |  |  |  |  |  |
| <= 60 | 135 | Reference |  |  | Reference |  |
| > 60 | 153 | 0.969 (0.533 - 1.762) | 0.917 |  | 0.781 (0.256 - 2.381) | 0.663 |
| SLC38A2 | 290 |  |  |  |  |  |
| Low | 145 | Reference |  |  | Reference |  |
| High | 145 | 1.005 (0.555 - 1.821) | 0.987 |  | 0.861 (0.247 - 3.005) | 0.814 |

**14. LAML**

| Characteristics | Total(N) | Univariate analysis | |  | Multivariate analysis | |
| --- | --- | --- | --- | --- | --- | --- |
|  |  | Hazard ratio (95% CI) | P value |  | Hazard ratio (95% CI) | P value |
| Gender | 139 |  |  |  |  |  |
| Female | 62 | Reference |  |  | Reference |  |
| Male | 77 | 1.024 (0.671 - 1.564) | 0.912 |  | 0.874 (0.567 - 1.349) | 0.544 |
| Age | 139 |  |  |  |  |  |
| <= 60 | 78 | Reference |  |  | Reference |  |
| > 60 | 61 | 3.321 (2.156 - 5.116) | **< 0.001** |  | 3.338 (2.142 - 5.201) | **< 0.001** |
| SLC38A2 | 139 |  |  |  |  |  |
| Low | 71 | Reference |  |  | Reference |  |
| High | 68 | 0.755 (0.493 - 1.157) | 0.197 |  | 0.897 (0.582 - 1.383) | 0.623 |

**15. LGG**

| Characteristics | Total(N) | Univariate analysis | |  | Multivariate analysis | |
| --- | --- | --- | --- | --- | --- | --- |
|  |  | Hazard ratio (95% CI) | P value |  | Hazard ratio (95% CI) | P value |
| WHO grade | 468 |  |  |  |  |  |
| G2 | 223 | Reference |  |  | Reference |  |
| G3 | 245 | 3.023 (2.022 - 4.519) | **< 0.001** |  | 2.066 (1.339 - 3.187) | **0.001** |
| IDH status | 527 |  |  |  |  |  |
| WT | 97 | Reference |  |  | Reference |  |
| Mut | 430 | 0.184 (0.129 - 0.263) | **< 0.001** |  | 0.227 (0.148 - 0.347) | **< 0.001** |
| Gender | 530 |  |  |  |  |  |
| Female | 238 | Reference |  |  | Reference |  |
| Male | 292 | 1.112 (0.791 - 1.563) | 0.542 |  | 1.380 (0.930 - 2.047) | 0.110 |
| Age | 530 |  |  |  |  |  |
| <= 40 | 265 | Reference |  |  | Reference |  |
| > 40 | 265 | 2.898 (2.015 - 4.168) | **< 0.001** |  | 2.725 (1.784 - 4.164) | **< 0.001** |
| SLC38A2 | 530 |  |  |  |  |  |
| Low | 265 | Reference |  |  | Reference |  |
| High | 265 | 1.122 (0.797 - 1.579) | 0.509 |  | 0.897 (0.605 - 1.330) | 0.589 |

**16. LIHC**

| Characteristics | Total(N) | Univariate analysis | |  | Multivariate analysis | |
| --- | --- | --- | --- | --- | --- | --- |
|  |  | Hazard ratio (95% CI) | P value |  | Hazard ratio (95% CI) | P value |
| Pathologic T stage | 370 |  |  |  |  |  |
| T1 | 183 | Reference |  |  | Reference |  |
| T2 | 94 | 1.428 (0.901 - 2.264) | 0.129 |  | 1.535 (0.849 - 2.775) | 0.157 |
| T3&T4 | 93 | 2.949 (1.982 - 4.386) | **< 0.001** |  | 3.238 (1.963 - 5.341) | **< 0.001** |
| Pathologic M stage | 272 |  |  |  |  |  |
| M0 | 268 | Reference |  |  | Reference |  |
| M1 | 4 | 4.077 (1.281 - 12.973) | **0.017** |  | 1.910 (0.557 - 6.555) | 0.303 |
| Gender | 373 |  |  |  |  |  |
| Female | 121 | Reference |  |  | Reference |  |
| Male | 252 | 0.793 (0.557 - 1.130) | 0.200 |  | 0.925 (0.584 - 1.465) | 0.738 |
| Age | 373 |  |  |  |  |  |
| <= 60 | 177 | Reference |  |  | Reference |  |
| > 60 | 196 | 1.205 (0.850 - 1.708) | 0.295 |  | 1.191 (0.767 - 1.851) | 0.436 |
| SLC38A2 | 373 |  |  |  |  |  |
| Low | 187 | Reference |  |  | Reference |  |
| High | 186 | 1.134 (0.802 - 1.604) | 0.477 |  | 1.065 (0.683 - 1.660) | 0.781 |

**17. LUAD**

| Characteristics | Total(N) | Univariate analysis | |  | Multivariate analysis | |
| --- | --- | --- | --- | --- | --- | --- |
|  |  | Hazard ratio (95% CI) | P value |  | Hazard ratio (95% CI) | P value |
| Pathologic T stage | 527 |  |  |  |  |  |
| T1 | 176 | Reference |  |  | Reference |  |
| T2 | 285 | 1.507 (1.059 - 2.146) | **0.023** |  | 1.555 (0.981 - 2.464) | 0.060 |
| T3&T4 | 66 | 3.095 (1.967 - 4.868) | **< 0.001** |  | 2.761 (1.554 - 4.904) | **< 0.001** |
| Pathologic N stage | 514 |  |  |  |  |  |
| N0 | 345 | Reference |  |  | Reference |  |
| N1 | 96 | 2.293 (1.632 - 3.221) | **< 0.001** |  | 1.978 (1.330 - 2.941) | **< 0.001** |
| N2&N3 | 73 | 2.993 (2.057 - 4.354) | **< 0.001** |  | 2.446 (1.578 - 3.791) | **< 0.001** |
| Pathologic M stage | 381 |  |  |  |  |  |
| M0 | 356 | Reference |  |  | Reference |  |
| M1 | 25 | 2.176 (1.272 - 3.722) | **0.005** |  | 1.691 (0.935 - 3.059) | 0.082 |
| Gender | 530 |  |  |  |  |  |
| Female | 283 | Reference |  |  | Reference |  |
| Male | 247 | 1.087 (0.816 - 1.448) | 0.569 |  | 0.952 (0.681 - 1.332) | 0.774 |
| Age | 520 |  |  |  |  |  |
| <= 65 | 257 | Reference |  |  | Reference |  |
| > 65 | 263 | 1.216 (0.910 - 1.625) | 0.186 |  | 1.233 (0.878 - 1.731) | **0.026** |
| SLC38A2 | 530 |  |  |  |  |  |
| Low | 267 | Reference |  |  | Reference |  |
| High | 263 | 1.292 (0.968 - 1.722) | 0.082 |  | 1.340 (0.951 - 1.889) | 0.094 |

**18. LUSC**

| Characteristics | Total(N) | Univariate analysis | |  | Multivariate analysis | |
| --- | --- | --- | --- | --- | --- | --- |
|  |  | Hazard ratio (95% CI) | P value |  | Hazard ratio (95% CI) | P value |
| Pathologic T stage | 496 |  |  |  |  |  |
| T1 | 114 | Reference |  |  | Reference |  |
| T2 | 289 | 1.237 (0.872 - 1.753) | 0.233 |  | 1.037 (0.702 - 1.531) | 0.856 |
| T3&T4 | 93 | 1.931 (1.277 - 2.920) | **0.002** |  | 1.416 (0.883 - 2.272) | 0.149 |
| Pathologic N stage | 490 |  |  |  |  |  |
| N0 | 317 | Reference |  |  | Reference |  |
| N1 | 128 | 1.076 (0.786 - 1.473) | 0.647 |  | 0.963 (0.674 - 1.377) | 0.837 |
| N2&N3 | 45 | 1.383 (0.887 - 2.158) | 0.152 |  | 1.615 (0.993 - 2.626) | 0.053 |
| Pathologic M stage | 415 |  |  |  |  |  |
| M0 | 408 | Reference |  |  | Reference |  |
| M1 | 7 | 3.112 (1.272 - 7.616) | **0.013** |  | 2.320 (0.841 - 6.395) | 0.104 |
| Gender | 496 |  |  |  |  |  |
| Female | 130 | Reference |  |  | Reference |  |
| Male | 366 | 1.211 (0.879 - 1.669) | 0.241 |  | 1.439 (0.990 - 2.093) | 0.057 |
| Age | 490 |  |  |  |  |  |
| <= 65 | 190 | Reference |  |  | Reference |  |
| > 65 | 300 | 1.279 (0.960 - 1.704) | 0.093 |  | 1.498 (1.083 - 2.073) | **0.015** |
| SLC38A2 | 496 |  |  |  |  |  |
| Low | 247 | Reference |  |  | Reference |  |
| High | 249 | 0.912 (0.695 - 1.197) | 0.508 |  | 0.871 (0.641 - 1.183) | 0.377 |

**19. MESO**

| Characteristics | Total(N) | Univariate analysis | |  | Multivariate analysis | |
| --- | --- | --- | --- | --- | --- | --- |
|  |  | Hazard ratio (95% CI) | P value |  | Hazard ratio (95% CI) | P value |
| Pathologic T stage | 84 |  |  |  |  |  |
| T1 | 14 | Reference |  |  | Reference |  |
| T2 | 25 | 0.977 (0.470 - 2.032) | 0.950 |  | 0.892 (0.368 - 2.164) | 0.800 |
| T3 | 32 | 1.056 (0.519 - 2.149) | 0.880 |  | 1.026 (0.422 - 2.494) | 0.955 |
| T4 | 13 | 0.775 (0.327 - 1.838) | 0.563 |  | 0.675 (0.206 - 2.214) | 0.517 |
| Pathologic N stage | 82 |  |  |  |  |  |
| N0 | 44 | Reference |  |  | Reference |  |
| N1 | 10 | 0.919 (0.443 - 1.907) | 0.820 |  | 0.880 (0.288 - 2.689) | 0.822 |
| N2&N3 | 28 | 0.869 (0.511 - 1.478) | 0.604 |  | 0.554 (0.255 - 1.204) | 0.136 |
| Pathologic M stage | 60 |  |  |  |  |  |
| M0 | 57 | Reference |  |  | Reference |  |
| M1 | 3 | 1.856 (0.441 - 7.817) | 0.399 |  | 2.078 (0.455 - 9.483) | 0.345 |
| Gender | 86 |  |  |  |  |  |
| Female | 16 | Reference |  |  | Reference |  |
| Male | 70 | 0.888 (0.494 - 1.595) | 0.691 |  | 0.579 (0.277 - 1.212) | 0.147 |
| Age | 86 |  |  |  |  |  |
| <= 65 | 46 | Reference |  |  | Reference |  |
| > 65 | 40 | 1.325 (0.826 - 2.125) | 0.243 |  | 1.534 (0.825 - 2.855) | 0.177 |
| SLC38A2 | 86 |  |  |  |  |  |
| Low | 43 | Reference |  |  | Reference |  |
| High | 43 | 1.198 (0.746 - 1.925) | 0.455 |  | 1.355 (0.703 - 2.611) | 0.364 |

**20. OV**

| Characteristics | Total(N) | Univariate analysis | |  | Multivariate analysis | |
| --- | --- | --- | --- | --- | --- | --- |
|  |  | Hazard ratio (95% CI) | P value |  | Hazard ratio (95% CI) | P value |
| Clinical stage | 376 |  |  |  |  |  |
| Stage I&Stage II | 24 | Reference |  |  | Reference |  |
| Stage III | 294 | 2.058 (0.911 - 4.649) | 0.083 |  | 1.639 (0.672 - 3.999) | 0.278 |
| Stage IV | 58 | 2.556 (1.085 - 6.025) | **0.032** |  | 1.663 (0.652 - 4.240) | 0.287 |
| Tumor status | 337 |  |  |  |  |  |
| Tumor free | 72 | Reference |  |  | Reference |  |
| With tumor | 265 | 9.598 (4.487 - 20.532) | **< 0.001** |  | 9.481 (4.425 - 20.313) | **< 0.001** |
| Age | 379 |  |  |  |  |  |
| <= 60 | 207 | Reference |  |  | Reference |  |
| > 60 | 172 | 1.352 (1.045 - 1.749) | **0.022** |  | 1.334 (1.013 - 1.757) | **0.040** |

**21. PAAD**

| Characteristics | Total(N) | Univariate analysis | |  | Multivariate analysis | |
| --- | --- | --- | --- | --- | --- | --- |
|  |  | Hazard ratio (95% CI) | P value |  | Hazard ratio (95% CI) | P value |
| Pathologic T stage | 177 |  |  |  |  |  |
| T1&T2 | 31 | Reference |  |  | Reference |  |
| T3 | 143 | 2.056 (1.090 - 3.878) | **0.026** |  | 0.840 (0.301 - 2.342) | 0.739 |
| T4 | 3 | 1.091 (0.140 - 8.489) | 0.934 |  | 0.000 (0.000 - Inf) | 0.997 |
| Pathologic N stage | 174 |  |  |  |  |  |
| N0 | 50 | Reference |  |  | Reference |  |
| N1 | 124 | 2.161 (1.287 - 3.627) | **0.004** |  | 1.733 (0.758 - 3.960) | 0.192 |
| Pathologic M stage | 85 |  |  |  |  |  |
| M0 | 80 | Reference |  |  | Reference |  |
| M1 | 5 | 0.773 (0.185 - 3.227) | 0.724 |  | 1.013 (0.225 - 4.558) | 0.986 |
| Gender | 179 |  |  |  |  |  |
| Female | 80 | Reference |  |  | Reference |  |
| Male | 99 | 0.813 (0.541 - 1.222) | 0.319 |  | 1.180 (0.606 - 2.298) | 0.626 |
| Age | 179 |  |  |  |  |  |
| <= 65 | 94 | Reference |  |  | Reference |  |
| > 65 | 85 | 1.285 (0.853 - 1.937) | 0.230 |  | 0.995 (0.517 - 1.912) | 0.987 |
| SLC38A2 | 179 |  |  |  |  |  |
| Low | 89 | Reference |  |  | Reference |  |
| High | 90 | 1.601 (1.060 - 2.417) | **0.025** |  | 1.192 (0.602 - 2.363) | **0.014** |

**22. PCPG**

| Characteristics | Total(N) | Univariate analysis | |  | Multivariate analysis | |
| --- | --- | --- | --- | --- | --- | --- |
|  |  | Hazard ratio (95% CI) | P value |  | Hazard ratio (95% CI) | P value |
| Gender | 184 |  |  |  |  |  |
| Female | 102 | Reference |  |  | Reference |  |
| Male | 82 | 4.431 (0.866 - 22.660) | 0.074 |  | 6.217 (1.098 - 35.184) | **0.039** |
| Age | 184 |  |  |  |  |  |
| <= 50 | 109 | Reference |  |  | Reference |  |
| > 50 | 75 | 2.823 (0.672 - 11.849) | 0.156 |  | 4.365 (0.900 - 21.167) | 0.067 |
| SLC38A2 | 184 |  |  |  |  |  |
| Low | 92 | Reference |  |  | Reference |  |
| High | 92 | 0.921 (0.230 - 3.694) | 0.907 |  | 1.443 (0.322 - 6.477) | 0.632 |

**23. PRAD**

| Characteristics | Total(N) | Univariate analysis | |  | Multivariate analysis | |
| --- | --- | --- | --- | --- | --- | --- |
|  |  | Hazard ratio (95% CI) | P value |  | Hazard ratio (95% CI) | P value |
| Pathologic T stage | 494 |  |  |  |  |  |
| T2 | 189 | Reference |  |  | Reference |  |
| T3 | 294 | 3.380 (0.631 - 18.091) | 0.155 |  | 2.962 (0.428 - 20.495) | 0.271 |
| T4 | 11 | 0.000 (0.000 - Inf) | 0.998 |  | 0.000 (0.000 - Inf) | 0.999 |
| Pathologic N stage | 428 |  |  |  |  |  |
| N0 | 348 | Reference |  |  | Reference |  |
| N1 | 80 | 3.470 (0.767 - 15.695) | 0.106 |  | 3.589 (0.730 - 17.653) | 0.116 |
| Clinical M stage | 460 |  |  |  |  |  |
| M0 | 457 | Reference |  |  | Reference |  |
| M1 | 3 | 59.773 (6.563 - 544.434) | **< 0.001** |  | 0.000 (0.000 - Inf) | 1.000 |
| Age | 501 |  |  |  |  |  |
| <= 60 | 225 | Reference |  |  | Reference |  |
| > 60 | 276 | 1.578 (0.441 - 5.650) | 0.484 |  | 2.620 (0.607 - 11.303) | 0.197 |
| SLC38A2 | 501 |  |  |  |  |  |
| Low | 250 | Reference |  |  | Reference |  |
| High | 251 | 1.240 (0.342 - 4.495) | 0.744 |  | 0.659 (0.156 - 2.781) | 0.570 |

**24. READ**

| Characteristics | Total(N) | Univariate analysis | |  | Multivariate analysis | |
| --- | --- | --- | --- | --- | --- | --- |
|  |  | Hazard ratio (95% CI) | P value |  | Hazard ratio (95% CI) | P value |
| Pathologic T stage | 164 |  |  |  |  |  |
| T1&T2 | 37 | Reference |  |  | Reference |  |
| T3 | 113 | 1.137 (0.372 - 3.470) | 0.822 |  | 0.572 (0.166 - 1.966) | 0.375 |
| T4 | 14 | 4.301 (1.147 - 16.129) | **0.031** |  | 2.322 (0.483 - 11.169) | 0.293 |
| Pathologic N stage | 162 |  |  |  |  |  |
| N0 | 84 | Reference |  |  | Reference |  |
| N1 | 45 | 2.400 (0.834 - 6.906) | 0.105 |  | 1.985 (0.577 - 6.828) | 0.276 |
| N2 | 33 | 3.683 (1.390 - 9.762) | **0.009** |  | 4.874 (1.440 - 16.502) | **0.011** |
| Pathologic M stage | 149 |  |  |  |  |  |
| M0 | 126 | Reference |  |  | Reference |  |
| M1 | 23 | 3.412 (1.424 - 8.174) | **0.006** |  | 2.093 (0.754 - 5.815) | 0.156 |
| Gender | 166 |  |  |  |  |  |
| Female | 75 | Reference |  |  | Reference |  |
| Male | 91 | 0.916 (0.422 - 1.988) | 0.824 |  | 0.694 (0.288 - 1.671) | 0.415 |
| Age | 166 |  |  |  |  |  |
| <= 65 | 82 | Reference |  |  | Reference |  |
| > 65 | 84 | 3.843 (1.535 - 9.622) | **0.004** |  | 11.300 (2.951 - 43.268) | **< 0.001** |
| SLC38A2 | 166 |  |  |  |  |  |
| Low | 83 | Reference |  |  | Reference |  |
| High | 83 | 0.650 (0.298 - 1.417) | 0.279 |  | 0.849 (0.329 - 2.192) | 0.735 |

**25. SARC**

| Characteristics | Total(N) | Univariate analysis | |  | Multivariate analysis | |
| --- | --- | --- | --- | --- | --- | --- |
|  |  | Hazard ratio (95% CI) | P value |  | Hazard ratio (95% CI) | P value |
| Gender | 263 |  |  |  |  |  |
| Female | 144 | Reference |  |  | Reference |  |
| Male | 119 | 0.905 (0.607 - 1.349) | 0.623 |  | 0.926 (0.619 - 1.385) | 0.708 |
| Age | 263 |  |  |  |  |  |
| <= 60 | 130 | Reference |  |  | Reference |  |
| > 60 | 133 | 1.285 (0.864 - 1.911) | 0.216 |  | 1.269 (0.850 - 1.894) | 0.244 |
| SLC38A2 | 263 |  |  |  |  |  |
| Low | 131 | Reference |  |  | Reference |  |
| High | 132 | 1.101 (0.742 - 1.635) | 0.633 |  | 1.060 (0.711 - 1.582) | 0.774 |

**26. SKCM**

| Characteristics | Total(N) | Univariate analysis | |  | Multivariate analysis | |
| --- | --- | --- | --- | --- | --- | --- |
|  |  | Hazard ratio (95% CI) | P value |  | Hazard ratio (95% CI) | P value |
| Pathologic T stage | 362 |  |  |  |  |  |
| T1 | 42 | Reference |  |  | Reference |  |
| T2 | 77 | 1.523 (0.826 - 2.806) | 0.178 |  | 1.826 (0.922 - 3.616) | 0.084 |
| T3 | 90 | 2.135 (1.179 - 3.867) | **0.012** |  | 2.932 (1.231 - 6.984) | **0.015** |
| T4 | 153 | 3.780 (2.109 - 6.776) | **< 0.001** |  | 5.791 (2.460 - 13.635) | **< 0.001** |
| Pathologic N stage | 403 |  |  |  |  |  |
| N0 | 225 | Reference |  |  | Reference |  |
| N1 | 73 | 1.503 (1.018 - 2.220) | **0.040** |  | 3.146 (1.061 - 9.331) | **0.039** |
| N2 | 49 | 1.540 (0.977 - 2.429) | 0.063 |  | 3.194 (1.046 - 9.749) | **0.041** |
| N3 | 56 | 2.744 (1.777 - 4.236) | **< 0.001** |  | 7.032 (2.319 - 21.322) | **< 0.001** |
| Pathologic stage | 411 |  |  |  |  |  |
| Stage I | 78 | Reference |  |  | Reference |  |
| Stage II | 140 | 1.600 (1.064 - 2.406) | **0.024** |  | 0.660 (0.315 - 1.379) | 0.269 |
| Stage III | 170 | 2.001 (1.356 - 2.954) | **< 0.001** |  | 0.368 (0.111 - 1.221) | 0.102 |
| Stage IV | 23 | 3.549 (1.798 - 7.008) | **< 0.001** |  | 0.700 (0.178 - 2.746) | 0.609 |
| Gender | 457 |  |  |  |  |  |
| Female | 173 | Reference |  |  | Reference |  |
| Male | 284 | 1.180 (0.885 - 1.574) | 0.261 |  | 1.070 (0.762 - 1.501) | 0.697 |
| Age | 457 |  |  |  |  |  |
| <= 60 | 247 | Reference |  |  | Reference |  |
| > 60 | 210 | 1.663 (1.256 - 2.201) | **< 0.001** |  | 1.158 (0.827 - 1.623) | 0.393 |
| SLC38A2 | 457 |  |  |  |  |  |
| Low | 225 | Reference |  |  | Reference |  |
| High | 232 | 0.857 (0.653 - 1.123) | 0.263 |  | 0.874 (0.632 - 1.209) | 0.417 |

**27. STAD**

| Characteristics | Total(N) | Univariate analysis | |  | Multivariate analysis | |
| --- | --- | --- | --- | --- | --- | --- |
|  |  | Hazard ratio (95% CI) | P value |  | Hazard ratio (95% CI) | P value |
| Pathologic T stage | 362 |  |  |  |  |  |
| T1&T2 | 96 | Reference |  |  | Reference |  |
| T3 | 167 | 1.713 (1.103 - 2.660) | **0.016** |  | 1.363 (0.842 - 2.204) | 0.207 |
| T4 | 99 | 1.729 (1.061 - 2.819) | **0.028** |  | 1.258 (0.722 - 2.190) | 0.418 |
| Pathologic N stage | 352 |  |  |  |  |  |
| N0 | 107 | Reference |  |  | Reference |  |
| N1 | 97 | 1.629 (1.001 - 2.649) | **0.049** |  | 1.429 (0.845 - 2.416) | 0.183 |
| N2 | 74 | 1.655 (0.979 - 2.797) | 0.060 |  | 1.516 (0.874 - 2.628) | 0.139 |
| N3 | 74 | 2.709 (1.669 - 4.396) | **< 0.001** |  | 2.456 (1.449 - 4.162) | **< 0.001** |
| Pathologic M stage | 352 |  |  |  |  |  |
| M0 | 327 | Reference |  |  | Reference |  |
| M1 | 25 | 2.254 (1.295 - 3.924) | **0.004** |  | 2.420 (1.335 - 4.388) | **0.004** |
| Gender | 370 |  |  |  |  |  |
| Female | 133 | Reference |  |  | Reference |  |
| Male | 237 | 1.267 (0.891 - 1.804) | 0.188 |  | 1.436 (0.982 - 2.101) | 0.062 |
| Age | 367 |  |  |  |  |  |
| <= 65 | 163 | Reference |  |  | Reference |  |
| > 65 | 204 | 1.620 (1.154 - 2.276) | **0.005** |  | 1.857 (1.289 - 2.676) | **< 0.001** |
| SLC38A2 | 370 |  |  |  |  |  |
| Low | 186 | Reference |  |  | Reference |  |
| High | 184 | 1.241 (0.893 - 1.725) | 0.199 |  | 1.076 (0.757 - 1.529) | 0.684 |

**28. TGCT**

| Characteristics | Total(N) | Univariate analysis | |  | Multivariate analysis | |
| --- | --- | --- | --- | --- | --- | --- |
|  |  | Hazard ratio (95% CI) | P value |  | Hazard ratio (95% CI) | P value |
| Pathologic T stage | 138 |  |  |  |  |  |
| T1 | 80 | Reference |  |  | Reference |  |
| T2 | 52 | 0.426 (0.033 - 5.487) | 0.513 |  | 0.000 (0.000 - Inf) | 1.000 |
| T3 | 6 | 8.322 (0.707 - 97.917) | 0.092 |  | 0.000 (0.000 - Inf) | 1.000 |
| Pathologic N stage | 64 |  |  |  |  |  |
| N0 | 51 | Reference |  |  | Reference |  |
| N1 | 11 | 0.000 (0.000 - Inf) | 1.000 |  | 0.359 (0.000 - Inf) | 1.000 |
| N2 | 2 | 0.000 (0.000 - Inf) | 1.000 |  | 0.117 (0.000 - Inf) | 1.000 |
| Pathologic M stage | 124 |  |  |  |  |  |
| M0 | 120 | Reference |  |  | Reference |  |
| M1 | 4 | 0.000 (0.000 - Inf) | 0.999 |  | 697784740.3409 (0.000 - Inf) | 1.000 |
| Age | 139 |  |  |  |  |  |
| <= 30 | 67 | Reference |  |  | Reference |  |
| > 30 | 72 | 0.382 (0.038 - 3.816) | 0.412 |  | 1332516025.5294 (0.000 - Inf) | 1.000 |
| SLC38A2 | 139 |  |  |  |  |  |
| Low | 69 | Reference |  |  | Reference |  |
| High | 70 | 0.674 (0.091 - 4.979) | 0.699 |  | 0.000 (0.000 - Inf) | 1.000 |

**29. THCA**

| Characteristics | Total(N) | Univariate analysis | |  | Multivariate analysis | |
| --- | --- | --- | --- | --- | --- | --- |
|  |  | Hazard ratio (95% CI) | P value |  | Hazard ratio (95% CI) | P value |
| Pathologic T stage | 510 |  |  |  |  |  |
| T1 | 143 | Reference |  |  | Reference |  |
| T2 | 169 | 1.007 (0.168 - 6.052) | 0.994 |  | 0.867 (0.052 - 14.439) | 0.921 |
| T3&T4 | 198 | 3.015 (0.665 - 13.678) | 0.153 |  | 2.265 (0.245 - 20.938) | 0.471 |
| Pathologic N stage | 462 |  |  |  |  |  |
| N0 | 229 | Reference |  |  | Reference |  |
| N1 | 233 | 1.405 (0.458 - 4.308) | 0.552 |  | 1.309 (0.266 - 6.443) | 0.740 |
| Pathologic M stage | 295 |  |  |  |  |  |
| M0 | 286 | Reference |  |  | Reference |  |
| M1 | 9 | 4.258 (0.909 - 19.952) | 0.066 |  | 2.700 (0.289 - 25.207) | 0.383 |
| Gender | 512 |  |  |  |  |  |
| Female | 373 | Reference |  |  | Reference |  |
| Male | 139 | 1.982 (0.717 - 5.478) | 0.187 |  | 0.743 (0.146 - 3.780) | 0.720 |
| Age | 512 |  |  |  |  |  |
| <= 45 | 243 | Reference |  |  | Reference |  |
| > 45 | 269 | 786840135.2007 (0.000 - Inf) | 0.997 |  | 604468116.1404 (0.000 - Inf) | 0.998 |
| SLC38A2 | 512 |  |  |  |  |  |
| Low | 256 | Reference |  |  | Reference |  |
| High | 256 | 0.808 (0.301 - 2.171) | 0.673 |  | 1.691 (0.440 - 6.495) | 0.444 |

**30. THYM**

| Characteristics | Total(N) | Univariate analysis | |  | Multivariate analysis | |
| --- | --- | --- | --- | --- | --- | --- |
|  |  | Hazard ratio (95% CI) | P value |  | Hazard ratio (95% CI) | P value |
| Gender | 119 |  |  |  |  |  |
| Female | 56 | Reference |  |  | Reference |  |
| Male | 63 | 0.620 (0.166 - 2.322) | 0.478 |  | 0.596 (0.156 - 2.282) | 0.450 |
| Age | 119 |  |  |  |  |  |
| <= 60 | 61 | Reference |  |  | Reference |  |
| > 60 | 58 | 4.038 (0.834 - 19.558) | 0.083 |  | 4.167 (0.862 - 20.153) | 0.076 |
| SLC38A2 | 119 |  |  |  |  |  |
| Low | 60 | Reference |  |  | Reference |  |
| High | 59 | 0.411 (0.100 - 1.681) | 0.216 |  | 0.395 (0.098 - 1.587) | 0.190 |

**31. UCEC**

| Characteristics | Total(N) | Univariate analysis | |  | Multivariate analysis | |
| --- | --- | --- | --- | --- | --- | --- |
|  |  | Hazard ratio (95% CI) | P value |  | Hazard ratio (95% CI) | P value |
| Clinical stage | 553 |  |  |  |  |  |
| Stage I | 342 | Reference |  |  | Reference |  |
| Stage II | 52 | 1.738 (0.833 - 3.626) | 0.140 |  | 1.758 (0.841 - 3.672) | 0.134 |
| Stage III | 130 | 3.084 (1.911 - 4.977) | **< 0.001** |  | 3.341 (2.056 - 5.428) | **< 0.001** |
| Stage IV | 29 | 8.082 (4.497 - 14.524) | **< 0.001** |  | 7.912 (4.388 - 14.266) | **< 0.001** |
| Age | 551 |  |  |  |  |  |
| <= 60 | 207 | Reference |  |  | Reference |  |
| > 60 | 344 | 1.850 (1.162 - 2.944) | **0.009** |  | 1.908 (1.194 - 3.049) | **0.007** |
| SLC38A2 | 553 |  |  |  |  |  |
| Low | 277 | Reference |  |  | Reference |  |
| High | 276 | 1.045 (0.696 - 1.567) | 0.833 |  | 0.827 (0.547 - 1.249) | 0.367 |

**32. UCS**

| Characteristics | Total(N) | Univariate analysis | |  | Multivariate analysis | |
| --- | --- | --- | --- | --- | --- | --- |
|  |  | Hazard ratio (95% CI) | P value |  | Hazard ratio (95% CI) | P value |
| Clinical stage | 57 |  |  |  |  |  |
| Stage I | 22 | Reference |  |  | Reference |  |
| Stage II | 5 | 1.030 (0.219 - 4.853) | 0.970 |  | 1.625 (0.314 - 8.411) | 0.563 |
| Stage III | 20 | 2.459 (1.084 - 5.575) | **0.031** |  | 3.303 (1.365 - 7.991) | **0.008** |
| Stage IV | 10 | 2.585 (0.949 - 7.037) | 0.063 |  | 2.395 (0.878 - 6.530) | 0.088 |
| Age | 57 |  |  |  |  |  |
| <= 65 | 22 | Reference |  |  | Reference |  |
| > 65 | 35 | 1.139 (0.571 - 2.273) | 0.711 |  | 1.052 (0.525 - 2.106) | 0.887 |
| SLC38A2 | 57 |  |  |  |  |  |
| Low | 28 | Reference |  |  | Reference |  |
| High | 29 | 0.619 (0.308 - 1.244) | 0.178 |  | 0.460 (0.205 - 1.033) | 0.060 |

**33. UVM**

| Characteristics | Total(N) | Univariate analysis | |  | Multivariate analysis | |
| --- | --- | --- | --- | --- | --- | --- |
|  |  | Hazard ratio (95% CI) | P value |  | Hazard ratio (95% CI) | P value |
| Pathologic M stage | 78 |  |  |  |  |  |
| M0 | 51 | Reference |  |  | Reference |  |
| M1 | 4 | 54.348 (5.517 - 535.347) | **< 0.001** |  | 56.899 (5.436 - 595.594) | **< 0.001** |
| MX | 23 | 0.521 (0.171 - 1.587) | 0.252 |  | 0.495 (0.161 - 1.523) | 0.220 |
| Gender | 80 |  |  |  |  |  |
| Female | 35 | Reference |  |  | Reference |  |
| Male | 45 | 1.542 (0.651 - 3.652) | 0.325 |  | 1.617 (0.613 - 4.263) | 0.332 |
| Age | 80 |  |  |  |  |  |
| <= 60 | 40 | Reference |  |  | Reference |  |
| > 60 | 40 | 2.123 (0.914 - 4.933) | 0.080 |  | 1.724 (0.691 - 4.301) | 0.243 |
| SLC38A2 | 80 |  |  |  |  |  |
| Low | 40 | Reference |  |  | Reference |  |
| High | 40 | 1.485 (0.624 - 3.532) | 0.371 |  | 1.616 (0.633 - 4.126) | 0.315 |
